# Quantum-confinement and defect-engineered N,S-carbon nanosheet–ruthenium quantum dot heterostructures for highly efficient organic solar cells

**DOI:** 10.1039/d6ra06453a

**Published:** 2026-08-11

**Authors:** Murali Balu, Thamilselvan A, Tholkappiyan Ramachandran, S. Sathiyaraj, Shanmugavel Chinnathambi, Natarajan Arumugam, Mohammad Ahmad Wadaan, Sandhanasamy Devanesan

**Affiliations:** a Centre for Applied Nanomaterials, Chennai Institute of Technology Kundrathur Chennai – 600 069 India muralib374@gmail.com +918124715364; b Basic Sciences-Chemistry, SR University Telangana-506371 India; c Department of Physics, College of Science, United Arab Emirates University P. O. Box 15551 Al-Ain, Abu Dhabi United Arab Emirates thols2006@gmail.com; d Department of Mechanical Engineering, Aarupadai Veedu Institute of Technology, Vinayaka Missions Research Foundation (DU) Chennai 603104 India; e Department of Chemistry, College of Science, King Saud University P.O. Box 2455 Riyadh 11451 Saudi Arabia; f Bioproducts Research Chair, Department of Zoology, College of Science, King Saud University P.O. Box 2455 Riyadh 11451 Saudi Arabia

## Abstract

The development of efficient and stable electron transport layers (ETLs) is essential for advancing high-performance organic solar cells (OSCs) by regulating interfacial charge dynamics and suppressing defect-mediated recombination. Herein, we develop a quantum-confinement and defect-engineered hybrid ETL based on porous nitrogen/sulfur co-doped carbon nanosheets decorated with ruthenium quantum dots (NSCNSs/RuQDs) for inverted OSCs. The hybrid material was synthesized *via* a facile one-pot solvothermal approach, enabling the uniform *in situ* growth of ultrasmall RuQDs (2–4 nm) on a conductive porous NSCNS framework. The strong RuQD–carbon coupling and heteroatom-induced electronic modulation generate abundant active sites, enhance interfacial charge interactions, and facilitate electron transport. Structural and spectroscopic investigations reveal that RuQD incorporation tailors the electronic structure of NSCNSs, broadens optical absorption, reduces defect-associated recombination, and induces quantum-confinement-driven bandgap modulation from 0.87 to 0.34 eV. Time-resolved photoluminescence measurements demonstrate an extended carrier lifetime of 5.44 ns for NSCNSs/RuQDs compared with 1.73 ns for pristine NSCNSs, confirming effective defect passivation and improved charge extraction. When integrated as a TiO_2_/NSCNSs/RuQDs bilayer ETL in PTB7-Th:PC71BM-based OSCs, the optimized devices achieve a power conversion efficiency (PCE) of 10.77 ± 0.57%, with a short-circuit current density of 23.18 ± 0.51 mA cm^−2^, open-circuit voltage of 0.83 ± 0.01 V, and fill factor of 0.56 ± 0.01, surpassing pristine NSCNS-based devices. Electrochemical impedance spectroscopy confirms reduced interfacial recombination, enhanced electron lifetime (85.61 ms), and improved charge collection efficiency (91.01%). This work highlights quantum-confined metal quantum dots integrated with heteroatom-engineered carbon frameworks as promising interfacial materials for next-generation optoelectronic and energy-conversion applications.

## Abstract 2

Ru quantum dot-decorated N,S-codoped carbon nanosheets, prepared by facile solvothermal route, serve as a multifunctional ETL. Oxygen vacancies enhance electron extraction, suppress recombination, enable 10.77% PCE in inverted organic solar cells.

## Graphical abstract

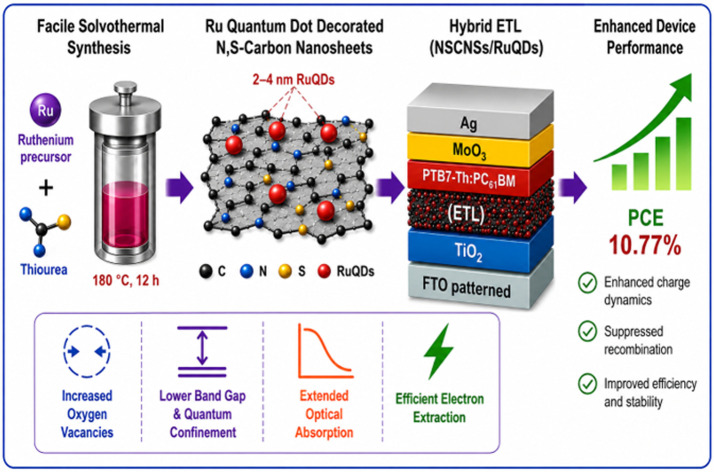

## 1 Introduction

Two-dimensional (2D) carbon nanosheets (CNSs) have emerged as a promising class of carbon allotropes owing to their exceptional physicochemical, electronic, and optical properties. Their broad light absorption, high chemical stability, low toxicity, and excellent biocompatibility make them attractive materials for advanced energy conversion and storage applications.^1,2^ Hence, they have great potential for many practical applications including, but not limited to, lithium-ion batteries,^3^ supercapacitors,^4^ organic solar cells,^5^ high-performance gas sensors,^6^ field-emitting materials,^7^ carbon dioxide adsorption,^8^ fuel cells,^9^ hydrogen storage,^10^ EDLC electrodes,^11^*etc.* To realize these applications, several new techniques have been developed for the synthesis of carbon nanosheets (CNs), including CVD,^12^ solvothermal technique,^13^ chemical/physical exfoliation,^14^ templating,^15^ and self-assembly method.^16^ In general, N–S-doped carbon nanosheets may be obtained by the thermal reaction between N-containing precursors such as cyanamide, dicyandiamide, melamine, thiourea, urea, and polyacrylamide.^17–21^ In addition, controllable carbon nanosheets (CNSs) can be synthesized through various approaches, including chemical and electrochemical exfoliation, chemical vapor deposition (CVD), carbonization, pyrolysis, self-assembly, and solvothermal methods. Owing to their high specific surface area, tunable pore structure, excellent electrical conductivity, and abundant active sites, these materials have found widespread applications in metal-ion sensing, field-emission devices, fuel cells, supercapacitors, and photocatalytic systems.^22,23^

Furthermore, carbon-based materials, including graphite, carbon nanosheets, graphene, and carbon nanotubes, have emerged as low-cost, solution-processable alternatives to conventional transparent conductive oxides (TCOs) and metal electrodes in organic solar cells (OSCs) because of their excellent electrical conductivity, chemical stability, mechanical flexibility, and compatibility with scalable fabrication techniques. Recent advances in carbon electrode engineering, synthesis strategies, and interface optimization have further highlighted their potential for developing efficient, stable, and cost-effective OSCs.^24^ Additionally, nitrogen- or boron-doped carbon-based nanostructures incorporated into the bulk heterojunction (BHJ) active layer effectively regulate charge transport by balancing electron and hole mobilities, suppressing space-charge accumulation and carrier recombination, enhancing charge extraction and collection efficiency, and consequently improving the power conversion efficiency (PCE) of organic solar cells.^25–27^ Current research highlights the development of N-doped porous two-dimensional (2D) carbon nanosheets with unique structural features, including large planar architectures, high specific surface areas, interconnected porous channels, excellent electrical conductivity, shortened ion diffusion pathways, and reduced transport resistance. These characteristics make them highly attractive as advanced carbon-based electrode materials, providing enhanced charge-transfer kinetics, improved rate capability, and prolonged cycling stability.^28,29^ In parallel with carbon-based architectures, Ru-based complexes and hybrid nanostructures are promising energy conversion materials due to their excellent light absorption, photostability, and charge-transfer properties. Their integration with carbon supports enhances interfacial electron transport and catalytic activity, enabling improved solar energy conversion and hydrogen evolution performance.^30,31^ Quantum dots (QDs) possess unique quantum confinement effects, tunable electronic properties, and abundant active sites, enabling their applications in energy conversion and storage. Ruthenium (Ru) and ruthenium oxide (RuO_2_) QDs exhibit excellent catalytic activity, conductivity, and redox behavior, making them promising materials for HER and supercapacitors. Coupling Ru-based QDs with carbon nanomaterials further improves electron transport, surface utilization, and structural stability for high-performance energy applications.^32,33^ In addition, hybrid materials composed of carbon nanomaterials and ruthenium quantum dots (RuQDs), particularly 2D carbon-based architectures, have attracted considerable attention because of their strong interfacial interactions and synergistic physicochemical properties. The combination of high surface area, excellent electrical conductivity, efficient light harvesting, rapid charge transfer, and structural stability enhances catalytic performance. In particular, RuQDs anchored on conductive 2D carbon nanosheets facilitate charge separation, shorten electron diffusion pathways, increase accessible active sites, and improve electrochemical stability, making them promising electrocatalysts for water splitting and other energy conversion applications.^34–36^ Recent advances in organic photovoltaics (OPVs) have been driven by the rational design of diverse donor and acceptor materials, enabling improved molecular packing, optimized bulk heterojunction morphology, and enhanced device performance toward 20% power conversion efficiency. Continued progress focuses on scalable fabrication, stability enhancement, and molecular engineering strategies for next-generation high-efficiency OPVs.^37,38^

The crystallinity and nanoscale morphology of the PTB7-Th:PC71BM bulk heterojunction (BHJ) active layer play critical roles in determining the photovoltaic performance of organic solar cells. The molecular ordering, phase separation, and donor–acceptor domain distribution strongly influence charge generation, electron and hole transport, and carrier recombination. Moreover, appropriate electron transport layers (ETLs) can improve the morphology and interfacial properties of PTB7-Th:PC71BM films, facilitating efficient charge extraction and suppressing recombination losses, thereby enhancing the overall power conversion efficiency and operational stability of the device.^39^ Solvent engineering is an effective approach for controlling bulk heterojunction (BHJ) morphology and photovoltaic performance in organic solar cells (OSCs). High-boiling-point solvent additives regulate film-formation kinetics by retarding solvent evaporation, enabling improved donor–acceptor phase separation, polymer chain ordering, and charge-transport pathway development.^40^ In this study, *N*-methyl-2-pyrrolidone (NMP) was introduced as a high-boiling-point solvent additive in the PTB7-Th:PC71BM active layer to regulate bulk heterojunction (BHJ) morphology through controlled solvent evaporation and selective solvation. NMP promotes favorable donor–acceptor phase separation, molecular organization, exciton dissociation, and charge transport, thereby reducing recombination losses. Meanwhile, the TiO_2_/NSCNSs/RuQDs electron transport layer (ETL) enhances interfacial charge extraction by improving energy-level alignment, passivating trap states, and suppressing recombination. Thus, the optimized photovoltaic performance results from the synergistic regulation of BHJ morphology and interfacial carrier transport. Solvent-additive engineering combined with interfacial ETL modification provides an effective strategy for improving organic solar cell performance by simultaneously optimizing active-layer morphology and charge-transfer processes.^41^

In this study, a novel porous hybrid nanostructure consisting of nitrogen- and sulfur-codoped carbon nanosheets (NSCNSs) decorated with ruthenium quantum dots (RuQDs) was successfully developed. The NSCNSs/RuQDs hybrid was synthesized through a modified solvothermal strategy, in which the incorporation of a ruthenium precursor into the carbonization process enabled the *in situ* nucleation and uniform anchoring of RuQDs onto the heteroatom-doped carbon nanosheet framework. The synergistic integration of porous N,S-codoped carbon nanosheets and highly dispersed RuQDs provides abundant active sites, enhanced light-harvesting capability, improved electrical conductivity, and efficient interfacial charge-transfer pathways. Furthermore, the electronic structure modulation induced by N,S heteroatom doping and RuQD incorporation contributes to improved optical absorption, accelerated carrier transport, and enhanced photoinduced electron injection. These unique structural and physicochemical characteristics make the NSCNSs/RuQDs hybrid a promising multifunctional material for advanced optoelectronic and photovoltaic applications. The results of optical characterization of NSCNSs/RuQDs confirm enhanced light absorption capability with a broad absorption peak. It corresponds to a decreased energy band gap, which was changed from 0.87 eV to 0.34 eV for NSCNSs/RuQDs. Confirmation was provided by the red shift of the absorption edge of the UV-vis diffuse reflectance spectrum. Additionally, tuning of the lattice optical properties in N- and S-doped CNSs/RuQDs leads to improved light harvesting capability, photosensitization efficiency, and better electron injection of photoinduced carriers. Subsequently, a TiO_2_/NSCNSs/RuQDs bilayer ETL was introduced into PTB7-Th:PC71BM-based BHJ OSCs to simultaneously optimize interfacial energy-level alignment, passivate trap states, enhance electron extraction, and suppress charge recombination while maintaining effective hole-blocking capability. As a result, the synergistic integration of BHJ morphological optimization and advanced interfacial modification enabled OSC devices with the architecture FTO/TiO_2_/NSCNSs/RuQDs/PTB7-Th:PC71BM/MoO_3_/Ag to achieve a remarkable photovoltaic performance with a short-circuit current density (*J*_SC_) of 23.18 ± 0.51 mA cm^−2^, open-circuit voltage (*V*_OC_) of 0.83 ± 0.01 V, fill factor (FF) of 0.56 ± 0.01, and power conversion efficiency (PCE) of 10.77 ± 0.57%. EIS proved the high charge recombination resistance, rapid charge transportation, prolonged electron lifetime, improved charge collection efficiency, and enhanced carrier lifetime.

## 2 Experimental section

### 2.1 Synthesis of porous N,S-carbon nanosheets (NSCNSs)

Porous N,S-codoped carbon nanosheets (NSCNSs) were synthesized *via* a solvothermal method. Briefly, 4.0 g of thiourea and 2.0 g of citric acid monohydrate were dissolved in 60 mL of DMF under magnetic stirring until a clear homogeneous solution was obtained. The precursor solution was transferred into a 100 mL Teflon-lined stainless-steel autoclave and heated at 180 °C for 12 h. After naturally cooling to room temperature, the black product was collected by centrifugation, thoroughly washed with deionized water and ethanol several times to remove residual organic species, and finally dried under vacuum at 80 °C for 12 h, yielding porous NSCNSs. A schematic illustration of the synthesis is presented in Fig. S1.

### 2.2. Synthesis of porous N,S-carbon nanosheets and ruthenium quantum dots (NSCNSs/RuQDs) hybrid structure

The NSCNSs/RuQDs hybrid was synthesized *via* a modified solvothermal method. Compared with the synthesis of pristine NSCNSs, the modification involved the introduction of a ruthenium precursor into the reaction system to facilitate the *in situ* formation and anchoring of Ru quantum dots (RuQDs) onto the developing N,S-codoped carbon nanosheet framework. Specifically, thiourea (4.0 g), citric acid monohydrate (2.0 g), and ruthenium(iii) chloride hydrate (RuCl_3_·*x*H_2_O, 0.30 g) were dissolved in 60 mL of *N*,*N*-dimethylformamide (DMF) under continuous stirring until a homogeneous precursor solution was obtained. The resulting solution was transferred into a Teflon-lined stainless-steel autoclave and maintained at 180 °C for 12 h. The Ru precursor content was optimized through preliminary experiments by varying the amount of RuCl_3_·*x*H_2_O while keeping the concentrations of thiourea and citric acid unchanged. The optimized Ru loading was selected based on the formation of uniformly dispersed RuQDs, suppression of Ru aggregation, and preservation of the porous N,S-codoped carbon nanosheet architecture. The optimized composition (0.30 g of RuCl_3_·*x*H_2_O) provided an appropriate density of Ru active sites while maintaining favorable structural characteristics.

After completion of the solvothermal reaction, the autoclave was naturally cooled to room temperature. The resulting reddish-black product was collected by centrifugation, repeatedly washed with deionized water and ethanol, and dried under vacuum at 80 °C overnight. During the solvothermal treatment, the Ru precursor underwent *in situ* conversion, resulting in the formation of ultrasmall metallic Ru quantum dots uniformly distributed and strongly coupled with the NSCNS framework. This process occurred without the addition of external reducing agents or stabilizing ligands. Therefore, the modified solvothermal approach enables the simultaneous formation of the N,S-codoped carbon nanosheet matrix and the *in situ* incorporation of RuQDs while maintaining the original solvothermal conditions used for NSCNS synthesis. A schematic illustration of the synthesis process is provided in Fig. S2. The Ru precursor loading was optimized through preliminary control experiments by varying the amount of RuCl_3_·*x*H_2_O while keeping the concentrations of thiourea and citric acid unchanged. Different Ru precursor concentrations were evaluated to regulate the nucleation and growth of Ru species on the carbon nanosheet framework. A lower Ru precursor concentration resulted in insufficient RuQD formation and a reduced density of active Ru sites, whereas excessive Ru precursor loading induced partial aggregation of Ru nanoparticles and compromised the uniform distribution of Ru species. The optimized Ru loading of 0.30 g RuCl_3_·*x*H_2_O provided an appropriate balance between RuQD nucleation, particle dispersion, and preservation of the porous nanosheet architecture. The resulting RuQDs exhibited a narrow size distribution of approximately 2–4 nm and were uniformly anchored on the NSCNS framework, as confirmed by HRTEM, XRD, and electrochemical analyses. Therefore, this optimized Ru precursor amount was employed for all subsequent synthesis experiments.

### 2.3 Fabrication of OSCs using porous NSCNSs/RuQDs hybrid nanostructures as the electron transport layer

**Fig. 1:**
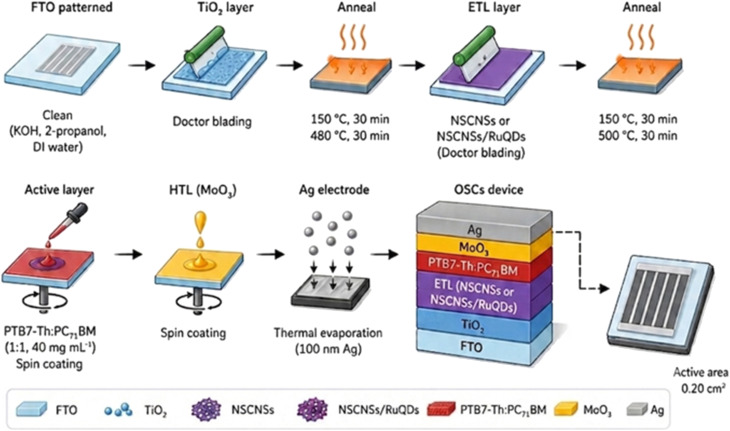
Schematic illustration of the fabrication of OSCs using porous NSCNSs and NSCNSs/RuQDs hybrid nanostructures as the electron transport layer.

The OSC devices have been fabricated with the following structure: FTO/TiO_2_/ETLs/PTB7-Th:PC71BM/MoO_3_/Ag. The preparation of ETLs based on porous NSCNSs and NSCNSs/RuQDs hybrids was carried out and applied in the fabrication of OSCs as follows. First, FTO glasses were patterned *via* chemical etching using Zn powder and HCl aqueous solution. Etched substrates were then treated with KOH and rinsed in 2-propanol and deionized water for 20 min each, followed by drying. In the first step, a layer of mesoporous TiO_2_ was deposited on the clean FTO substrate *via* the doctor-blading technique from TiO_2_ paste, which created an active cell area of 0.20 cm^2^. The deposited TiO_2_-coated FTO substrates were air-dried and thermally annealed successively at 150 °C for 30 min and at 480 °C for 30 min, followed by cooling down to ambient temperature. Second, different porous NSCNSs and NSCNSs/RuQDs hybrids were coated as an upper layer on the TiO_2_-coated FTO substrates, ensuring the same active area as the underlying layer by Scotch-taping the doctor-blade edge when depositing the ETL films. The prepared bilayer structures were air-dried, thermally annealed sequentially at 150 °C for 30 min and 500 °C for 30 min, followed by cooling down to ambient temperature. Third, the photoactive layer was produced *via* spin coating. The active layer based on a blend of PTB7-Th : PC71BM was synthesized in a 1 : 1 weight ratio at a total concentration of 40 mg mL^−1^ using a mixed solution of 1,2-dichlorobenzene-*N*-methyl-2-pyrrolidone (1 : 3 volume). The obtained blend solution was stirred at 45 °C for 24 h to dissolve completely. After the deposition of the PTB7-Th:PC71BM active layer, the substrate was moved to a nitrogen glove box. The solution was spin-coated at 2000 rpm for 30 s, creating a bulk heterojunction (BHJ) layer. The as-prepared film was thermally annealed at 150 °C for approximately 10 min to facilitate the dense formation of the BHJ structure. As HTM, MoO_3_ was coated at 3000 rpm for 30 s. Lastly, a 100 nm thick Ag cathode was evaporated *via* thermal evaporation under vacuum with a base pressure of 7 × 10^−7^ mbar. Fig. 1 shows the schematic illustration of the fabrication of OSCs using porous NSCNSs and NSCNSs/RuQDs hybrid nanostructures as the electron transport layer.

## 3 Results and discussion

### 3.1 Structural and vibrational analysis of NSCNSs/RuQDs hybrid nanostructures

**Fig. 2:**
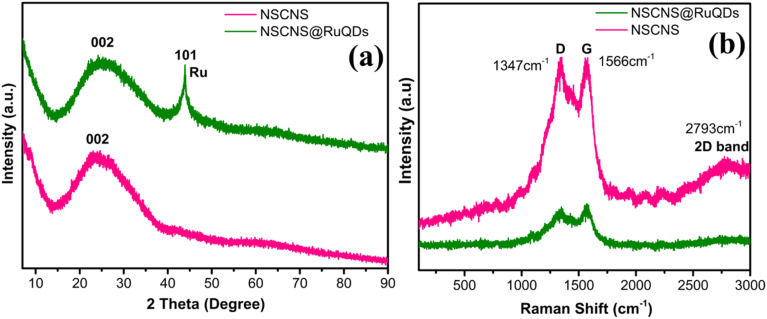
(a) XRD spectra of NSCNSs and NSCNSs/RuQDs, and (b) FT-IR spectra of NSCNSs and NSCNSs/RuQDs hybrid structure.

X-ray diffraction studies on both NSCNSs and NSCNSs/RuQDs hybrid structures have been done and are displayed in Fig. 2(a). It can be observed from the XRD pattern of both samples that there exists a broad peak at 2*θ* = 24.20°, representing the (002) peak of disordered or amorphous carbon structure with a graphitic lattice containing sp^2^ carbon. Moreover, to study the amorphous nature of the surface of NSCNSs and NSCNSs/RuQDs, more XRD measurements were performed and displayed in Fig. 4(a). This clearly shows that all the lattices present in the particles are defined, which means that the Ru quantum dots in the hybrid structure possess a single-crystal nature. Furthermore, the crystallographic nature of the synthesized Ru quantum dots is in excellent agreement with the X-ray diffraction (XRD) results obtained for the Ru quantum dot-decorated carbon nanosheets, as illustrated in Fig. 2(a). A well-defined diffraction peak centered at 2*θ* = 43.85° is observed, corresponding to an interplanar spacing (*d*-spacing) of approximately 2.05 Å. This reflection can be indexed to the (101) crystallographic plane of metallic Ru(0), consistent with the standard diffraction data for face-centered cubic (fcc) ruthenium (JCPDS Card No. 06-0663).^38,42^ The sharp and intense nature of this peak indicates the formation of highly crystalline Ru nanodomains and confirms the successful reduction of ruthenium species to the metallic state. Moreover, the absence of additional diffraction features attributable to ruthenium oxides or secondary phases highlights the phase purity of the synthesized quantum dots. The excellent correlation between the observed diffraction parameters and reference crystallographic data provides strong evidence for the well-defined fcc crystal structure and high structural integrity of the Ru quantum dots anchored on the carbon nanosheet NSCNSs/RuQDs framework.

Raman spectra for NSCNSs and NSCNSs/RuQDs are shown in Fig. 2(b). In all prepared materials, there were two bands, namely, the D band and G band. D band was found to be responsible for vibration modes of dangling bonds, while the G band was attributed to the in-plane vibrations of sp^2^ hybridization of carbon atoms. It was observed that the NSCNSs and NSCNSs/RuQDs exhibited the presence of the D band at 1347 cm^−1^ and G band at 1566 cm^−1^.^29,43–45^ These two bands are indicative of disordered carbon structures, wherein the bond distances involved C–C, C–N, and C–S. It must be noted that both NSCNSs and NSCNSs/RuQDs gave the same ratio of intensity *I*_D_/*I*_G_ of 0.86.^29^ This suggests that the crystalline structure obtained from the XRD analysis shows a significant relation to the red shift of the photoluminescence emission.

### 3.2 Surface functionalization and charge-carrier dynamics of NSCNSs/RuQDs hybrid nanostructures

**Fig. 3:**
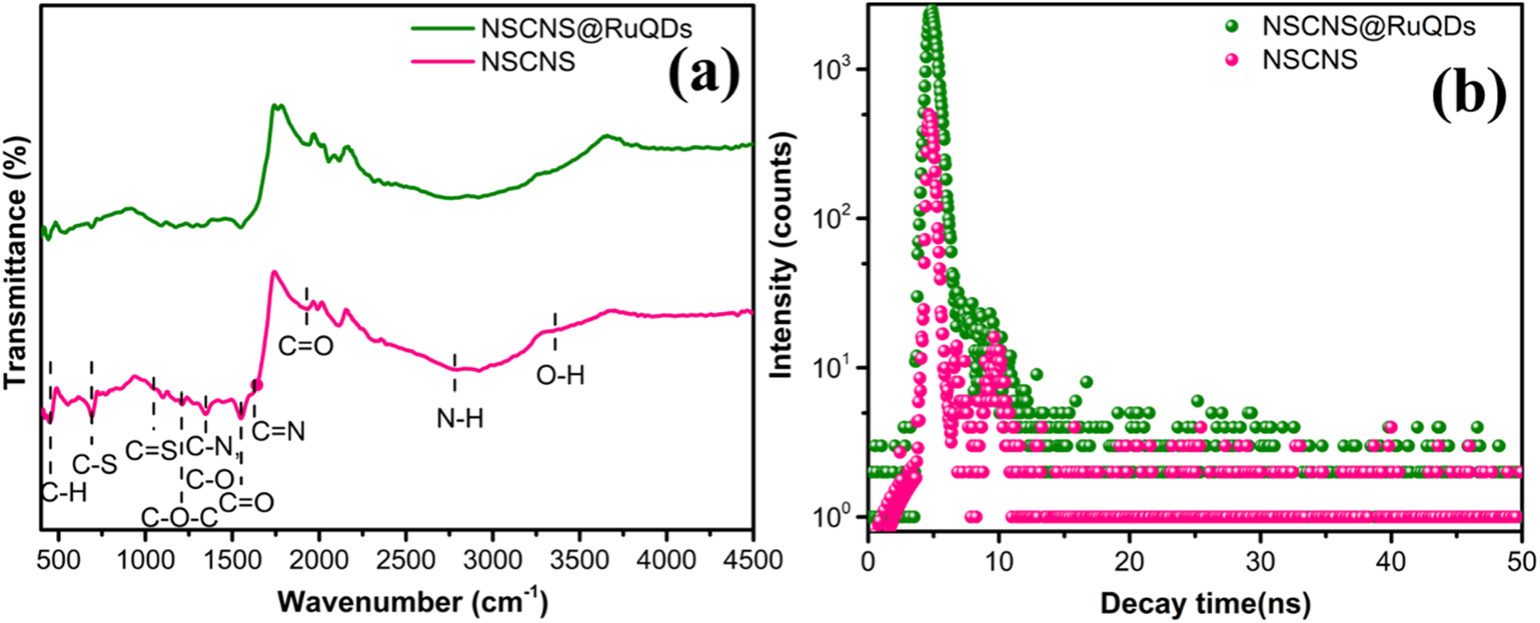
(a) FT-IR spectra of NSCNSs and NSCNSs/RuQDs, and (b) TRPL spectra of NSCNSs and NSCNSs/RuQDs hybrid structure.

FT-IR spectrum of NSCNSs and NSCNSs/RuQDs is shown in Fig. 3(a). This study aimed to use Fourier Transform-Infrared Spectroscopy for determining surface functional groups of both NSCNSs and NSCNSs/RuQDs structures. The result obtained from the IR spectra of both materials is shown in Fig. 3(a), which gives a better understanding of chemical reactions between materials by providing information about the existing chemical bonds in their surfaces. The IR bands located at 1384 cm^−1^, 1422 cm^−1^, and 1599 cm^−1^ are associated with C–H symmetric bending, CH_2_ bending, and N–H bending, respectively.^29,44–47^ The broad bands at 3516–2900 cm^−1^ indicate the presence of O–H and N–H stretching of carboxyl groups and amines, respectively, as the main functional groups on the surface of NSCNSs.^29,44–47^ Besides, IR spectra have shown peaks at 2858 cm^−1^ and 2931 cm^−1^ representing asymmetrical and symmetrical C–H stretching, respectively.^29,44–47^ Another small band at 2485 cm^−1^ indicates the presence of S–H stretching. The band located at 1734 cm^−1^ corresponds to C=O stretching vibrations, while the one at 1634 cm^−1^ involves C=C and C=N bonds. The in-plane bending vibrations of C–N bonds are associated with the band located at 1425 cm^−1^ (ref. 29 and 44–47). The prominent band at 1146 cm^−1^ indicates the presence of C–S bond vibrations, while the one at 1031 cm^−1^ is associated with C–O–C bending vibrations. Finally, the band at 860 cm^−1^ is associated with C–O stretching vibrations.

Time-resolved photoluminescence (TRPL) measurements were carried out to study photogenerated carrier dynamics within the NSCNSs and NSCNSs@RuQDs nanostructures, shown in Fig. 3(b). It is clear that the hybrid possesses the same PL emission peaks as pure NSCNSs, yet shows a much higher average lifetime, going from 1.73 ns for NSCNSs to 5.44 ns for NSCNSs@RuQDs. The extended lifetime signifies the inhibition of non-radiative recombination at the interface between acceptor and donor moieties, suggesting an efficient passivation of defect sites by Ru quantum dots (RuQDs). An extended lifetime corresponds to an energy barrier that prevents carrier recombination, and therefore, leads to an increase in the probability of their separation. More specifically, the increased lifetime of excitons in the hybrid structure is a result of less trap-related losses, meaning that carriers are in the free state and can be extracted as current. Additionally, a lifetime of 5.44 ns is a sign of the energy level alignment in the NSCNSs/RuQD heterojunction; it makes the reverse electron transfer process difficult while facilitating directional charge transport.

### 3.3 Morphological evolution and structural characterization of NSCNSs/RuQDs hybrid nanostructures

**Fig. 4:**
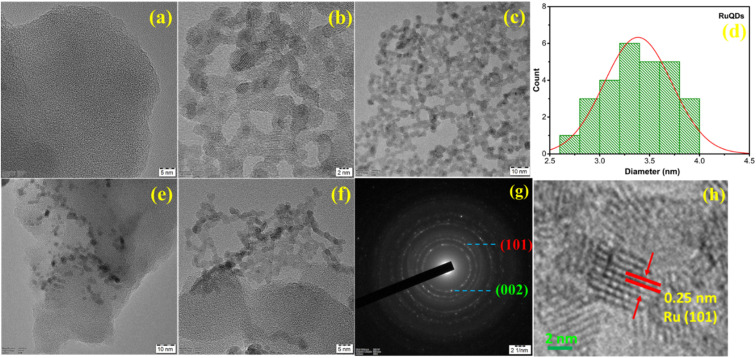
HRTEM images of NSCNSs and NSCNSs/RuQDs hybrid. (a) Pristine NSCNSs. (b and c) RuQDs. (d) RuQD size distribution. (e and f) RuQDs anchored on the NSCNS framework. (g) SAED pattern of NSCNSs/RuQDs showing graphitic carbon and metallic Ru diffraction rings. (h) Enlarged HRTEM image with Ru(101) lattice fringes.

To investigate the morphology, particle size, and microstructural characteristics of the synthesized porous N,S-codoped carbon nanosheets (NSCNSs) and the NSCNSs/RuQDs hybrid, high-resolution transmission electron microscopy (HRTEM) was employed, and the corresponding images are presented in Fig. 4. As shown in Fig. 4(a), the pristine NSCNSs possess a well-defined two-dimensional porous nanosheet architecture with a thin and wrinkled morphology, providing a high surface area suitable for anchoring metallic nanostructures. Such a porous framework is advantageous for maximizing the exposure of active sites and promoting efficient mass and electron transport.

The HRTEM images shown in Fig. 4(b) and (c) reveal that the synthesized Ru quantum dots (RuQDs) are nearly spherical and uniformly distributed, with particle diameters ranging from 2 to 4 nm. The corresponding particle size distribution histogram (Fig. 4(d)) yields an average particle diameter of approximately 3.4 nm, confirming the successful synthesis of ultrasmall RuQDs with a narrow size distribution. The uniform particle size suggests effective nucleation and controlled crystal growth during the one-pot solvothermal synthesis, which is beneficial for maximizing the quantum confinement effect and catalytic activity. The HRTEM images of the NSCNSs/RuQDs hybrid (Fig. 4e and f) clearly demonstrate that the Ru quantum dots (RuQDs) are uniformly dispersed and firmly anchored onto the porous NSCNS framework, without noticeable nanoparticle aggregation. The intimate interfacial contact between the metallic RuQDs and the conductive nitrogen–sulfur co-doped carbon nanosheets indicates the formation of strong interfacial interactions, which are expected to facilitate rapid electron transfer across the heterointerface and enhance structural stability during electrochemical operation. Furthermore, the HRTEM image in Fig. 4(f) reveals that the RuQDs are interconnected through chain-like assemblies, in which individual quantum dots remain in close proximity and establish nanoscale electronic connectivity.

The formation of these interconnected RuQD networks is closely associated with the synergistic regulation of nucleation, growth kinetics, and interparticle interactions during the solvothermal synthesis process. Such an interconnected architecture enables effective electronic coupling between neighboring RuQDs while preserving the intrinsic quantum confinement effects arising from their nanoscale dimensions. Compared with isolated quantum dots, the interconnected RuQD assemblies provide continuous charge-transfer pathways, improved electrical conductivity, and enhanced utilization of surface-accessible catalytic sites. In addition, the strong coupling between RuQDs and the conductive NSCNS substrate can further promote interfacial electron transport and prevent nanoparticle migration or aggregation during electrochemical reactions. Therefore, the unique combination of quantum confinement, interparticle electronic interactions, and robust RuQD–carbon coupling is expected to play a crucial role in enhancing the electrochemical activity and durability of the NSCNSs/RuQDs hybrid.

The selected-area electron diffraction (SAED) pattern (Fig. 4(g)) exhibits well-defined concentric diffraction rings with interplanar spacings of approximately 0.32 and 0.205 nm, which are indexed to the (002) plane of graphitic carbon and the (101) plane of metallic Ru(0), respectively. The simultaneous presence of these characteristic reflections confirms the successful crystalline incorporation of RuQDs within the porous NSCNS framework while preserving the structural integrity of the carbon support. Furthermore, the sharp and distinct diffraction rings indicate the high crystallinity of both constituent phases and strong structural coherence at the RuQDs/NSCNS interface, demonstrating the effective formation of the hybrid nanostructure.

Further structural analysis from the enlarged HRTEM image (Fig. 4(h)) reveals well-resolved and continuous lattice fringes extending across individual RuQDs, indicating their highly ordered atomic arrangement and single-crystalline nature. The measured lattice fringe spacing of approximately 0.205 nm corresponds to the (101) crystallographic plane of hexagonal metallic Ru(0), which is in excellent agreement with the XRD results (Fig. 2(a)). The close correlation between the HRTEM and XRD analyses confirms the formation of phase-pure, highly crystalline Ru quantum dots with a well-defined crystal structure. The high crystallinity and nanoscale structural coherence of the RuQDs are expected to facilitate efficient electron transport and enhance their intrinsic electrocatalytic activity.

The elemental composition and spatial distribution of the synthesized materials were investigated by energy-dispersive X-ray spectroscopy (EDS) elemental mapping (Fig. S3). As shown in Fig. S3(a), the pristine NSCNSs exhibit a homogeneous distribution of C, N, and S throughout the carbon nanosheets, confirming the successful co-doping of nitrogen and sulfur within the carbon framework. As illustrated in Fig. S3(b), the NSCNSs/RuQDs hybrid displays a uniform distribution of C, N, S, and Ru over the porous nanosheets, demonstrating the homogeneous incorporation of Ru quantum dots (RuQDs) without observable aggregation or phase segregation. These results, together with the HRTEM and SAED analyses, unequivocally confirm the successful fabrication of a highly crystalline NSCNSs/RuQDs hybrid with excellent structural integrity, compositional homogeneity, and strong interfacial coupling between the RuQDs and the N,S-co-doped carbon nanosheets.

### 3.4 Chemical state analysis and electronic structure modification of NSCNSs/RuQDs hybrid electron transport layers

**Fig. 5:**
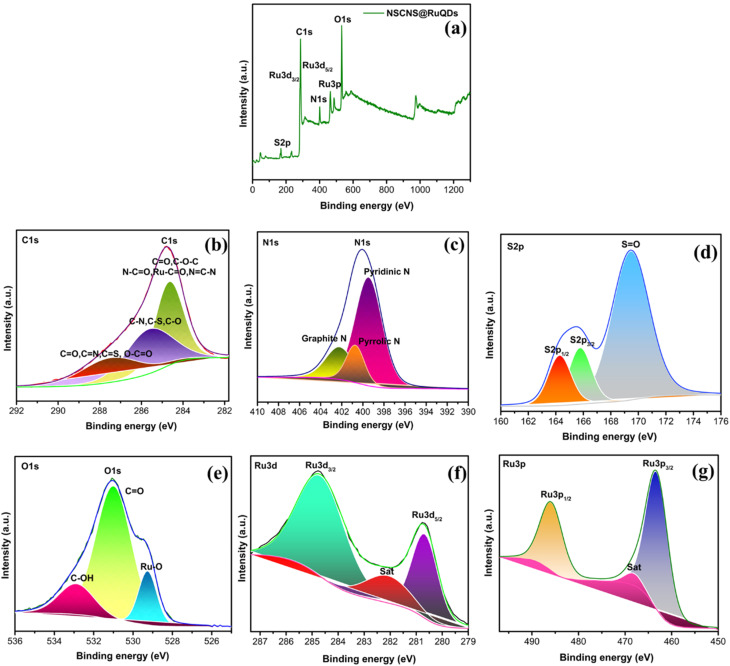
(a) Wide range of X-ray photoelectron spectra of C 1s, N 1s, O 1s, Si 2p, Ru 3d, and Ru 3p for the NSCNSs/RuQDs hybrid structure. (b) C 1s, (c) N 1s, (d) Si 2p, (e) O 1s, (f) Ru 3d, and (g) Ru 3p.

The chemical compositions and surface features of the NSCNSs/RuQDs system were identified using X-ray photoelectron spectroscopy (XPS). The wide-range survey spectrum in Fig. 5(a) indicated the existence of ruthenium (Ru), carbon (C), sulfur (S), nitrogen (N), and oxygen (O) in the sample. The survey spectrum in Fig. 5(a) showed binding energies at 164, 227, 284, 400, and 533 eV that correspond to S 1s, S 2p, C 1s, N 1s, and O 1s, respectively, revealing that nitrogen and sulfur exist in the material prepared by thiourea.^29,48–52,54^ High-resolution spectra of C 1s in Fig. 5(b) were analyzed to find different graphitic elements corresponding to various chemical bonds, including sp^2^ carbon atoms in graphene (C–C), C=C, C=O, C=N, C=S, and other chemical bonds. The graphitic elements revealed by high-resolution spectra of C 1s Fig. 5(b) were observed at 284.4, 285.5, and 286.8 eV and corresponding to sp^2^ C in graphene (C–C), C=C, C=O, C=N, C=S, O–C=O, as well as sp^3^ C in C–N, C–S, C–O, C–O–C, N–C=O, Ru–C=O, N=C–N, and C=O in carboxyl groups, respectively.^29,48–52,54^ Three peaks were found in the deconvoluted O 1s Fig. 5(c) spectra and assigned to O–Ru–O, C=O, C–O/C–OH/C–O–C, N–O, and S–O bonds. These results reveal that specific oxygen functionalities exist on the surface of the NSCNSs/RuQDs. Specifically, a peak at 531.02 eV represents adsorbed oxygen atoms associated with oxygen-vacancy sites on the surface of Ru, namely Ru–O.^29,48–52,54^

In addition, Fig. 5(d) verifies the existence of nitrogen in NSCNSs/RuQDs by the N 1s spectra. The N 1s signal was deconvoluted in Fig. 5(d), showing three peaks of nitrogen-containing groups, namely pyridinic N (399.48 eV), pyrrolic N (C–N–C) (400.73 eV), and graphitic N or N–H (402.27 eV).^29,48–52,54^ The high-resolution deconvoluted XPS spectrum of S 2p is shown in Fig. 5(e). This suggests the existence of C–S–C. Two peaks of C–S–C were found at 164.29 eV and 165.80 eV, corresponding to S 2p_3/2_ and S 2p_1/2_ of thiophene (C–S–C), respectively. In addition, there is one peak of the oxidized sulfur group (S=O) with (–C–S–Ox) bonding (*x* = 2–4) at 169.45 eV.^29,48–52,54^ Two peaks appeared in the high-resolution XPS spectrum of Ru 3d in Fig. 5(f), corresponding to Ru 3d_3/2_ and Ru 3d_5/2_ of metallic Ru at 285.49 eV and 281.44 eV, respectively. Curve fitting analysis of Ru 3d_5/2_ (Fig. 5(f)) confirmed that there are two spin-orbital doublets of Ru, along with two shake-up satellite peaks at 283.01 eV.^53–56^ Likewise, peaks in the high-resolution XPS spectrum of Ru 3p in Fig. 5(g) were found at 463.53 eV and 486.17 eV, representing Ru 3p_3/2_ and Ru 3p_1/2_ of metallic Ru, respectively.^53–56^ Curve fitting of Ru 3p_3/2_ (Fig. 5(f)) also verified the existence of two spin-orbital doublets of Ru, with two shake-up satellite peaks at 468.33 eV.^53–56^ In addition, the existence of oxygen vacancies is indicated by the low binding energy of some oxygen atoms. To enhance the efficiency of OSCs, a co-doping technique of metal (N, S, C, Ru) is applied in NSCNSs/RuQDs hybrids. The effect of co-doping with N and S elements together with Ru alters the electronic structure and the local environment. This produces defects and perturbations in order to increase the number of oxygen vacancies. On the contrary, other transition metals can also produce some energy levels in the bandgap of NSCNSs/RuQDs hybrids. This allows the electrons to occupy these energy levels. As a result, the number of oxygen vacancies increases. This change is more effective for OSCs, which require efficient charge separation and transportation in order to produce electrical current. The use of Ru-metal doping and the resulting increase in oxygen vacancies and charge carrier mobility in NSCNSs/RuQDs hybrids improves the performance of OSCs. This provides a significant increase in power conversion efficiency. Experimental results also suggest that NSCNSs/RuQDs hybrid-based ETLs increase oxygen vacancies. This leads to improved charge separation and *J*_sc_ and *V*_oc_ in OSCs.

### 3.5 RuQDs-induced optical bandgap modulation and enhanced light absorption properties of NSCNS-based electron transport layers

**Fig. 6:**
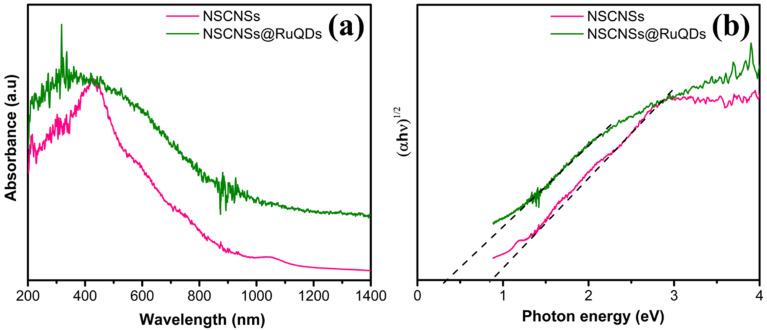
(a) The absorption spectra of individual nanostructures with light-absorbing properties, namely NSCNSs and NSCNSs/RuQDs, were examined. (b) The Tauc plots were created for each light-absorbing nanostructure, namely NSCNSs and NSCNSs/RuQDs.

To investigate the optical absorption properties of thin-film NSCNSs and NSCNSs/RuQDs samples, diffuse reflectance spectroscopy (DRS) measurements were taken within the UV-vis range. Optical absorption spectra for the ETL of NSCNSs and NSCNSs/RuQDs samples were performed to reveal any changes caused by the co-doping of Ru, N, S, and C in the ETLs of NSCNSs/RuQDs. In particular, optical absorption of the ETL materials was investigated in the near-IR region, namely, 200–1400 nm Fig. 6(a). The optical absorption of NSCNSs/RuQDs ETL is broader compared with that of the NSCNSs' ETL in the visible to near-IR spectral range. As expected, NSCNSs and NSCNSs/RuQDs samples demonstrate absorption bands centered at 200–520 nm, 587 nm, and 750 nm with an absorption edge at 520 nm. However, Fig. 6(a) demonstrates that the NSCNSs/RuQDs thin film has much absorption in the 200–1100 nm range, where an absorption edge at 1100 nm is revealed. The latter is linked to the presence of Ru in the NSCNSs thin films, which increases NSCNSs' energy levels just above the valence band, decreases their bandgap, and allows them to absorb in the visible to near-IR regions. In addition, the redshift of the absorption edge and increase in the 200–1100 nm absorption contribute to the increase in light absorption ability of NSCNSs/RuQDs compared with NSCNSs samples. The presence of Ru in NSCNSs transforms their color from dark gray to reddish-gray, which is due to an increase in the absorption coefficient of the NSCNSs/RuQDs hybrid in the whole visible region (200–1400 nm). The optical bandgaps of the pristine NSCNSs and NSCNSs/RuQDs hybrid were determined from UV-vis diffuse reflectance spectroscopy (DRS) using the Kubelka–Munk transformation and the Tauc relationship:

1 (*αhν*)^1/*n*^ = *A*(*hν* − *E*_g_)

where *α* is the absorption coefficient obtained from the Kubelka–Munk function, *hν* is the photon energy, *A* is a proportionality constant, *E*_g_ is the optical bandgap, and *n* denotes the nature of the optical transition. In this study, *n* = 2 was employed, corresponding to an indirect allowed transition. This selection is based on the intrinsic electronic structure of the synthesized N,S-codoped carbon nanosheets, which consist of partially graphitized carbon domains with abundant structural disorder, heteroatom-induced defects, and localized electronic states. These characteristics typically give rise to indirect optical transitions, as commonly observed in defect-rich carbon-based semiconducting materials.

The corresponding Tauc plots, obtained by plotting (*αhν*)^1/2^ as a function of photon energy, are shown in Fig. 6(b), while the complete linear fitting regions are provided in Fig. S4. The optical bandgaps were determined by extrapolating the linear portions of the absorption edge to the photon-energy axis. The pristine NSCNSs exhibited an optical bandgap of approximately 0.87 eV, whereas the NSCNSs/RuQDs hybrid displayed a significantly reduced optical bandgap of approximately 0.34 eV. The high linear correlation coefficients (*R*^2^ = 0.98 for NSCNSs and *R*^2^ = 0.95 for NSCNSs/RuQDs) confirm the reliability of the bandgap determination.

The pronounced reduction in the optical bandgap following RuQD incorporation indicates substantial modulation of the electronic structure of the N,S-codoped carbon framework. This behavior is attributed to the formation of Ru-induced interfacial electronic states, strong electronic coupling between the RuQDs and the carbon nanosheets, and an increased density of defect-associated states, all of which promote electronic transitions at lower photon energies. Consequently, the NSCNSs/RuQDs hybrid exhibits an extended absorption edge and enhanced visible-to-near-infrared light absorption, consistent with the DRS results. Although the reduced optical bandgap contributes to broader optical absorption, the primary role of the ultrathin NSCNSs/RuQDs layer in the photovoltaic device is to function as an efficient electron-transport and interfacial modification layer. Therefore, the enhanced device performance is predominantly attributed to improved interfacial electron extraction, accelerated charge-transfer kinetics, stronger electronic coupling, and suppressed charge recombination, rather than to direct photon harvesting by the electron-transport layer itself. The complete Tauc fitting regions presented in Fig. S4(a and b) further substantiate the reliability and reproducibility of the extracted optical bandgap values.

It is worth emphasizing that the NSCNSs/RuQDs hybrid material ETL ensures the control of the optical properties of the hybrid materials, leading to light absorption increase, enhancement of photosensitizer properties, and better light harvesting capability. In addition, the electron injection rate is optimized in this way. XPS analysis has confirmed the better creation of electron–hole pairs in NSCNSs/RuQDs as hybrid ETLs. The introduction of Ru into NSCNSs/RuQDs leads to more effective absorption and narrowing of the bandgap. Therefore, Ru incorporation leads to the formation of mid-gap energy levels, oxygen vacancy increase, and enhanced charge carrier transport.

### 3.6 Enhanced photovoltaic performance of inverted organic solar cells through RuQDs-modified NSCNS electron transport layers

The feasibility of employing nitrogen- and sulfur-co-doped carbon nanospheres (NSCNSs) and Ru quantum dot-decorated NSCNSs (NSCNSs/RuQDs) as electron transport layers (ETLs) in inverted organic solar cells (OSCs) was systematically investigated. The photovoltaic devices were fabricated with the architectures FTO/TiO_2_/NSCNSs/PTB7-Th:PC71BM/MoO_3_/Ag and FTO/TiO_2_/NSCNSs/RuQDs/PTB7-Th:PC71BM/MoO_3_/Ag, where NSCNSs and NSCNSs/RuQDs served as the ETL and hybrid ETL, respectively. Device performance was evaluated from current density–voltage (*J*–*V*) characteristics measured under simulated AM 1.5 G solar illumination (100 mW cm^−2^). To ensure statistical reliability and fabrication reproducibility, seven independently fabricated devices (*n* = 7) were characterized for each architecture, and all photovoltaic parameters are reported as mean ± standard deviation.

**Fig. 7:**
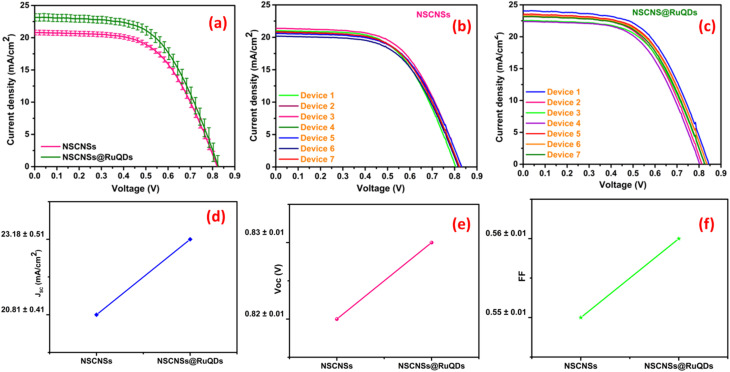
(a) Average current density–voltage (*J*–*V*) characteristics of organic solar cells incorporating pristine NSCNSs and NSCNSs/RuQDs electron transport layers under AM 1.5 G illumination (100 mW cm^−2^); error bars represent one standard deviation (*n* = 7 independent devices). (b and c) Statistical distributions of the photovoltaic parameters for OSCs based on NSCNSs and NSCNSs/RuQDs ETLs, respectively. (d–f) Comparison of the average short-circuit current density (*J*_SC_), open-circuit voltage (*V*_OC_), and fill factor (FF) with standard deviation error bars (*n* = 7).

Fig. 7(a) presents the average *J*–*V* characteristics of the OSCs employing pristine NSCNSs and NSCNSs/RuQDs ETLs, with error bars representing one standard deviation obtained from seven independent devices. The corresponding statistical distributions of the photovoltaic parameters are shown in Fig. 7(b and c), demonstrating narrow distributions and excellent device-to-device reproducibility. Fig. 7(d–f) compare the average values of the short-circuit current density (*J*_SC_), open-circuit voltage (*V*_OC_), and fill factor (FF), respectively, together with their standard deviations. The complete photovoltaic parameters are summarized in Table 1. Fig. 7(a) compares the average *J*–*V* characteristics of OSCs employing pristine NSCNSs and NSCNSs/RuQDs hybrid electron transport layers (ETLs), with error bars representing one standard deviation obtained from seven independent devices. The statistical distributions of the photovoltaic parameters for the pristine NSCNSs and NSCNSs/RuQDs devices are presented in Fig. 7(b) and (c), respectively, confirming excellent device-to-device reproducibility with narrow performance distributions. Furthermore, Fig. 7(d)–(f) compare the average values of *J*_SC_, *V*_OC_, and FF, respectively, including standard deviation error bars.

**Table 1:** Photovoltaic parameters are reported as mean ± standard deviation obtained from seven independently fabricated devices (*n* = 7) for each device configuration

Photoanode	*J*_SC_ (mA cm^−2^)	*V*_OC_ (V)	FF	PCE (%)
FTO/TiO_2_/NSCNSs/PTB7-Th:PC71BM/MoO_3_/Ag	20.81 ± 0.41	0.82 ± 0.01	0.55 ± 0.01	9.39 ± 0.47
FTO/TiO_2_/NSCNSs/RuQDs/PTB7-Th:PC71BM/MoO_3_/Ag	23.18 ± 0.51	0.83 ± 0.01	0.56 ± 0.01	10.77 ± 0.57

The photovoltaic parameters obtained from the seven independent devices are summarized in Table 1. The OSC incorporating the pristine NSCNS ETL exhibited an average *J*_SC_ of 20.81 ± 0.41 mA cm^−2^, *V*_OC_ of 0.82 ± 0.01 V, FF of 0.55 ± 0.01, and power conversion efficiency (PCE) of 9.39 ± 0.47%. Upon incorporation of the NSCNSs/RuQDs hybrid ETL, the corresponding photovoltaic parameters increased to *J*_SC_ of 23.18 ± 0.51 mA cm^−2^, *V*_OC_ of 0.83 ± 0.01 V, FF of 0.56 ± 0.01, and PCE of 10.77 ± 0.57%, respectively. The relatively small standard deviations obtained for all photovoltaic parameters confirm excellent fabrication reproducibility and demonstrate that the observed performance enhancement originates from the Ru-modified ETL rather than device-to-device variation.

The simultaneous enhancement of *J*_SC_, *V*_OC_, FF, and PCE indicates that incorporation of RuQDs effectively improves the interfacial charge-transport properties of the TiO_2_/NSCNS bilayer. Among these parameters, the most pronounced improvement is observed in *J*_SC_, suggesting more efficient photogenerated charge collection. This enhancement is attributed primarily to improved electron extraction, reduced interfacial recombination, and enhanced carrier transport at the ETL/active-layer interface rather than changes in the intrinsic photoactive layer. These conclusions are fully consistent with the electrochemical impedance spectroscopy (EIS) results, which reveal reduced charge-transfer resistance, prolonged electron lifetime, and increased charge-collection efficiency following Ru incorporation. Fig. S5 shows the fabrication of OSCs using porous NSCNSs and NSCNSs/RuQDs hybrid nanostructures as the electron transport layer. Power conversion efficiency (PCE), along with other parameters, of the studied OSCs is shown in Table 1.

UV-visible absorption measurements demonstrate that the NSCNSs/RuQDs hybrid exhibits broad optical absorption over the 200–1100 nm wavelength range. Although Ru incorporation decreases the optical bandgap of the ETL from approximately 0.87 to 0.34 eV and enhances its optical absorption, the TiO_2_/NSCNSs/RuQDs layer is an ultrathin interfacial film whose contribution to total photon absorption is negligible compared with that of the substantially thicker PTB7-Th:PC71BM active layer. Consequently, the enhanced photovoltaic performance cannot be attributed primarily to additional photon harvesting within the ETL. Instead, Ru incorporation modifies the electronic properties of the ETL by improving electrical conductivity, optimizing interfacial energy-level alignment, and passivating defect states, thereby facilitating electron extraction while suppressing trap-assisted recombination.

The enhanced *J*_SC_ is therefore primarily associated with improved carrier collection efficiency rather than increased optical absorption by the ETL itself. Chemical interactions between the TiO_2_ nanoparticles and the NSCNSs/RuQDs hybrid further facilitate electron injection into the TiO_2_ electron transport network, accelerating charge separation and reducing interfacial charge accumulation. These improvements contribute directly to the observed increase in photocurrent.

Recent studies have shown that quantum-confinement engineering within active-layer materials can reduce energy losses by regulating charge-transfer dynamics and suppressing non-radiative recombination through favorable quantum-well-like energy configurations.^57^ In contrast, the present work adopts an interfacial engineering strategy by incorporating Ru quantum dots into the ETL rather than modifying the photoactive layer. The primary role of the NSCNSs/RuQDs hybrid is therefore to regulate the electrode/active-layer interface by improving electron extraction, optimizing interfacial energetics, passivating defects, and suppressing charge recombination. Accordingly, the enhanced device performance originates predominantly from improved interfacial carrier transport instead of modifications of exciton dissociation or bulk energy-loss mechanisms within the active layer. These two approaches should therefore be regarded as complementary strategies for improving photovoltaic performance, and their combination may provide an attractive route toward further efficiency enhancement.

Although Ru incorporation decreases the optical bandgap of the NSCNS-based ETL, this reduction does not compromise its electron-selective transport characteristics. Carrier selectivity is governed primarily by energy-level alignment and interfacial transport properties rather than by the optical bandgap alone. The TiO_2_/NSCNSs/RuQDs bilayer maintains efficient electron extraction while preserving a sufficient energetic barrier for hole transport. Simultaneously, Ru incorporation enhances electrical conductivity and passivates interfacial trap states, thereby suppressing trap-assisted recombination. The increased recombination resistance, prolonged electron lifetime, and enhanced charge-collection efficiency obtained from EIS measurements, together with the simultaneous improvements in *J*_SC_, *V*_OC_, FF, and PCE, confirm that the Ru-modified ETL promotes efficient carrier selectivity without inducing detrimental hole leakage.

Although the incorporation of RuQDs slightly modifies the optical properties of the TiO_2_-based electron transport layer (ETL), including a broadened absorption response and reduced apparent optical bandgap. Although a narrower ETL bandgap may theoretically introduce parasitic absorption losses because incident light passes through the ETL before reaching the active layer, this effect is expected to be minimal in the present device configuration. The TiO_2_/NSCNSs/RuQDs ETL is employed as an ultrathin interfacial layer with a thickness of only several tens of nanometers, which is considerably thinner than the BHJ active layer. Therefore, the fraction of incident photons absorbed by the ETL remains negligible, allowing sufficient photon flux to reach the photoactive layer. Furthermore, the enhanced electronic functionality provided by Ru incorporation dominates over any minor optical losses associated with the modified ETL. The introduction of RuQDs improves interfacial electron extraction, enhances charge transport pathways, and suppresses trap-assisted recombination at the ETL/active-layer interface. These improvements are supported by EIS analysis, which reveals reduced charge-transfer resistance, increased carrier lifetime, and more efficient charge collection in the optimized devices. Accordingly, the optimized TiO_2_/NSCNSs/RuQDs-based OSCs exhibit enhanced *J*_SC_, FF, *V*_OC_, and PCE values compared with the reference device. The increased photocurrent density further confirms that parasitic absorption within the ultrathin ETL does not represent a dominant loss mechanism. Instead, the improved photovoltaic performance primarily originates from more efficient interfacial carrier extraction and reduced recombination losses enabled by the Ru-modified ETL architecture.

Based on the above discussion, reducing the bandgap of active-layer materials enables improved energy-level alignment and more efficient charge separation between donor and acceptor components. Furthermore, Ru incorporation into the NSCNSs/RuQDs hybrid ETL, combined with bandgap optimization, can suppress charge recombination losses, as evidenced by photoluminescence (PL) analysis. Enhanced charge separation reduces electron–hole recombination before charge collection, thereby facilitating photocurrent generation and improving PCE. Therefore, Ru-based interfacial engineering of NSCNSs/RuQDs ETLs and bandgap modulation represent effective strategies for enhancing carrier transport and photovoltaic performance.

Fig. 7(e) shows that the FTO/TiO_2_/NSCNSs/RuQDs/PTB7-Th:PC71BM/MoO_3_/Ag device exhibits a slightly higher open-circuit voltage (*V*_OC_) than the corresponding device employing the pristine NSCNS ETL. The enhancement in *V*_OC_ is primarily attributed to improved interfacial charge extraction and suppressed non-radiative recombination at the ETL/active-layer interface following Ru incorporation, rather than to the reduction in the optical bandgap of the ETL itself. The incorporation of RuQDs into the NSCNS matrix significantly improves the photovoltaic performance of OSCs by facilitating electron extraction, optimizing interfacial energy-level alignment, reducing charge recombination losses, and improving carrier transport across the electron transport layer. These results demonstrate the effectiveness of the NSCNSs/RuQDs hybrid ETL in enhancing charge-transfer properties and photovoltaic device performance. The improved interfacial characteristics, including reduced charge accumulation and suppressed recombination pathways, may provide favorable conditions for improving the durability of OSC devices during operation. To evaluate the reproducibility of the photovoltaic performance, seven independently fabricated devices (*n* = 7) for each configuration were measured under identical fabrication and testing conditions. The photovoltaic parameters, including short-circuit current density (*J*_SC_), open-circuit voltage (*V*_OC_), fill factor (FF), and power conversion efficiency (PCE), are summarized as average values with corresponding standard deviations in Table 1. The statistical analysis demonstrates that the NSCNSs/RuQDs-based devices exhibit consistently improved photovoltaic performance compared with the pristine NSCNS-based devices, confirming the reliability and reproducibility of the enhanced electron transport characteristics induced by RuQD incorporation. These effects reduce charge accumulation and recombination losses, leading to more efficient quasi-Fermi-level splitting and a modest increase in *V*_OC_. This interpretation is consistent with the electrochemical impedance spectroscopy (EIS) results, which demonstrate reduced charge-transfer resistance, prolonged electron lifetime, and enhanced charge-collection efficiency after Ru incorporation.

As shown in Fig. 7(f), the OSC based on the TiO_2_/NSCNSs/RuQDs hybrid ETL exhibits a slightly enhanced fill factor (FF) compared with the pristine NSCNS-based device. This improvement is attributed to more efficient interfacial electron extraction, reduced series resistance, and suppressed trap-assisted recombination, leading to improved carrier transport and charge collection. The uniformly distributed RuQDs on the NSCNS surface provide additional electron-transfer pathways while maintaining the electron-selective characteristics of the ETL. As a result, the TiO_2_/NSCNSs/RuQDs bilayer promotes faster electron transport and minimizes interfacial losses, contributing to simultaneous enhancements in *J*_SC_, *V*_OC_, FF, and PCE. The average PCE increases from 9.39 ± 0.47% for the pristine NSCNS-based device to 10.77 ± 0.57% after RuQDs modification, demonstrating the effectiveness of RuQDs as an interfacial engineering strategy for improving OSC performance.

The statistical PCE distribution of OSCs employing NSCNSs and NSCNSs/RuQDs ETLs (Fig. S6) further confirms the reproducibility and reliability of the performance enhancement. In addition, the MoO_3_ hole transport layer facilitates efficient hole extraction and favorable interfacial energy alignment, reducing charge accumulation and recombination losses. The synergistic integration of the TiO_2_/NSCNSs/RuQDs ETL and MoO_3_ HTL establishes efficient charge-selective transport pathways, resulting in improved charge collection, reduced interfacial losses, and enhanced photovoltaic performance. These findings demonstrate that RuQD modification of NSCNS-based ETLs is an effective approach for optimizing interfacial charge transport in inverted OSCs.

Collectively, the incorporation of the TiO_2_/NSCNSs/RuQDs bilayer effectively improves interfacial charge extraction and minimizes carrier transport losses during device operation. The optimized device exhibits a *J*_SC_ value of approximately 23.2 mA cm^−2^, which is among the highest values reported for PTB7-Th:PC71BM fullerene-based OSCs employing interfacial engineering approaches. Similar enhancements in photocurrent density have been reported for fullerene-based OSCs through the introduction of optimized electron transport layers, where improved energy-level alignment, enhanced electron selectivity, and reduced interfacial recombination contribute to more efficient carrier collection.^58–60^ To confirm the reliability of the photovoltaic measurements, all *J*–*V* characteristics were recorded under calibrated AM 1.5 G illumination (100 mW cm^−2^) using a solar simulator calibrated with a certified silicon reference cell. In addition, the active device area was precisely defined using a shadow mask to minimize measurement errors associated with device-area determination. Repeated measurements from independently fabricated devices showed consistent photovoltaic performance, confirming the reliability and reproducibility of the obtained *J*_SC_ values.

Although non-fullerene acceptor (NFA)-based systems currently dominate the highest-performance OSC technologies, the PTB7-Th:PC71BM fullerene-based bulk heterojunction was selected in this study as a well-established model platform to investigate the intrinsic influence of electron transport layer (ETL) modification. The relatively mature understanding of PTB7-Th:PC71BM in terms of energy-level alignment, charge transport, and morphology-performance relationships provides an effective framework for isolating the effects of interfacial engineering without additional complexity arising from the morphological evolution and energetic disorder frequently encountered in NFA-based systems. The primary focus of this work is the development of a Ru-modified NSCNSs ETL rather than the optimization of the photoactive layer. The TiO_2_/NSCNSs/RuQDs bilayer architecture improves interfacial electron extraction by providing additional conductive pathways, facilitating charge transfer, and suppressing interfacial recombination. Consequently, the enhanced *J*_SC_, FF, PCE, carrier lifetime, and charge-collection efficiency observed in the optimized devices are mainly attributed to the improved interfacial properties of the ETL. The effectiveness of this interfacial strategy is not restricted to fullerene-based OSCs. The fundamental mechanisms associated with improved electron selectivity, reduced charge-transfer resistance, and enhanced carrier extraction are also highly relevant to modern NFA-based devices. Therefore, the TiO_2_/NSCNSs/RuQDs ETL represents a potentially versatile interfacial engineering approach, and its integration with high-performance NFA systems will be explored in future studies.

Overall, the TiO_2_/NSCNSs/RuQDs hybrid ETL significantly enhances photovoltaic performance by simultaneously improving electron extraction, reducing interfacial recombination, increasing carrier lifetime, and promoting efficient charge collection. These synergistic improvements result in an increase in PCE from 9.39 ± 0.47% to 10.77 ± 0.57%, demonstrating that Ru quantum dot modification of NSCNS-based ETLs constitutes an effective interfacial engineering strategy for high-performance inverted organic solar cells.

### 3.7 Interfacial charge transport and recombination dynamics in NSCNSs/RuQDs-modified electron transport layers

**Fig. 8:**
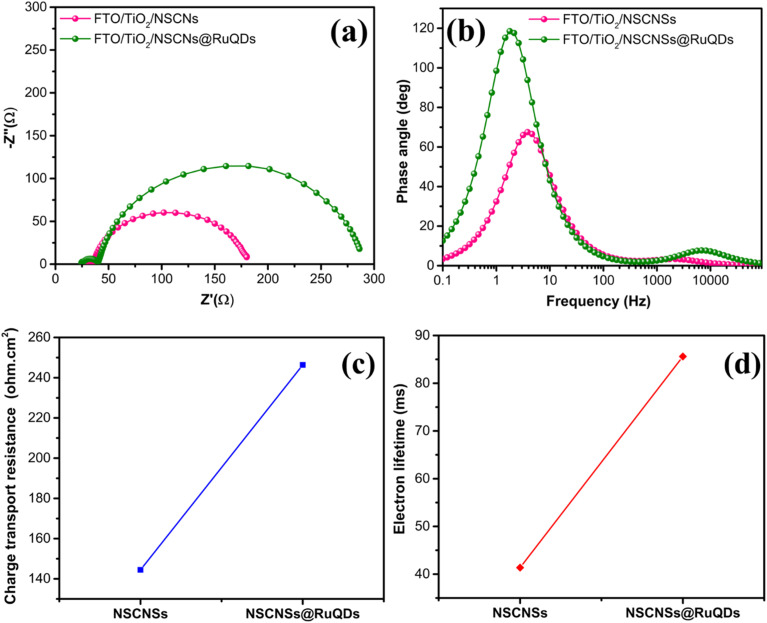
Electrochemical impedance spectroscopy (EIS) of OSCs of BHJ device with FTO/TiO_2_/NSCNSs/PTB7-Th:PC71BM/MoO_3_/Ag, and FTO/TiO_2_/NSCNSs/RuQDs/PTB7-Th:PC71BM/MoO_3_/Ag light absorbing nanostructures recorded at open circuit voltage (−0.8 V): (a) Nyquist plots, (b) Bode phase plots, (c) charge recombination resistances and (d) electron lifetime.

EIS was performed on the two sets of OSCs:FTO/TiO_2_/NSCNSs/PTB7-Th:PC71BM/MoO_3_/Ag and FTO/TiO_2_/NSCNSs/RuQDs/PTB7-Th:PC71BM/MoO_3_/Ag BHJ devices. Here, the properties of electron transport resistance of the BHJ device (*R*_t_), the chemical capacitance (*C*_µ_) of the BHJ device, and effective electron lifetime (*τ*_n_) are analyzed based on the bias voltage of −0.8 V at the active layer/ETL interface. Parameters *R*_t_, *C*_µ_, and *τ*_n_ can be extracted by fitting the experimental data of EIS to the equivalent circuit model, illustrated in Fig. 8. It helps us understand more about the impedance behavior and extract useful information from the experiment. Moreover, the EIS parameters for OSCs constructed using FTO/TiO_2_/NSCNSs/PTB7-Th:PC71BM/MoO_3_/Ag and FTO/TiO_2_/NSCNSs/RuQDs/PTB7-Th:PC71BM/MoO_3_/Ag BHJ devices, which involve different photo-absorbing nanostructured ETLs, were summarized in Fig. S7. The fitting parameters were derived from the fitting results of the Nyquist plot acquired at a bias voltage of −0.8 V with the equivalent circuit in Fig. S7.

It has been established from the Nyquist plots obtained through EIS analysis for the organic solar cells (OSCs), including the FTO/TiO_2_/NSCNSs/PTB7-Th:PC71BM/MoO_3_/Ag and FTO/TiO_2_/NSCNSs/RuQDs/PTB7-Th:PC71BM/MoO_3_/Ag bulk heterojunctions (BHJ), that there are two semicircles in the high frequency range (100 kHz–10 Hz), see Fig. 8(a). The high-frequency semicircle refers to the electrochemical behavior due to charge transfer at the interface between the counter electrode (as a hole transport layer, HTL) and the active layer. The intermediate frequency semicircle refers to electrochemical behaviors associated with the charge transfer resistance at the interface between the electron transport layer (ETL; FTO/TiO_2_/ETLs) and the active layer. The EIS studies carried out with the applied voltage of −0.8 V showed that larger semicircles, in the intermediate frequency regime, show significant alteration depending upon the inclusion of Ru species in the NSCNSs/RuQDs lattice. The presence of Ru metal causes alteration in the photon absorption capacity of the ETLs. As evident from Fig. 8(a) and (c), charge transport resistances of OSCs with various BHJs vary from 246.37 Ω cm^2^ to 144.47 Ω cm^2^ as we move from FTO/TiO_2_/NSCNSs/RuQDs/PTB7-Th:PC71BM/MoO_3_/Ag to FTO/TiO_2_/NSCNSs/PTB7-Th:PC71BM/MoO_3_/Ag, which indicates marked enhancement in charge recombination resistance for OSCs with the FTO/TiO_2_/NSCNSs/RuQDs/PTB7-Th:PC71BM/MoO_3_/Ag device. Fig. 8(b) gives the Bode phase plots of the BHJ devices present in the OSCs. The characteristic frequency (*f*_max_) calculated from the Bode phase plot is used to calculate the electron lifetime (*τ*_n_) of the OSCs using

2 *τ*_n_ = 1/(2π*f*_max_)

From Fig. 8(b) and Table 2, it is evident that the OSCs with the FTO/TiO_2_/NSCNSs/RuQDs/PTB7-Th:PC71BM/MoO_3_/Ag BHJ possess significantly increased electron lifetime (85.61 ms) as compared with the OSCs having the FTO/TiO_2_/NSCNSs/PTB7-Th:PC71BM/MoO_3_/Ag BHJ device (41.36 ms). In general, an increased value of charge transport resistance results in a longer electron lifetime (*τ*_n_) for OSCs. Furthermore, it can be seen from Fig. 8(b) and (d) that enhanced electron lifetime has been achieved with FTO/TiO_2_/NSCNSs/RuQDs/PTB7-Th:PC71BM/MoO_3_/Ag devices owing to the 0D/1D nanoparticle/nanorod configuration, which increases the electron mean free paths. Consequently, the FTO/TiO_2_/NSCNSs/RuQDs/PTB7-Th:PC71BM/MoO_3_/Ag devices show higher electron lifetimes as compared with the FTO/TiO_2_/NSCNSs/PTB7-Th:PC71BM/MoO_3_/Ag organic solar cells.

**Table 2:** EIS parameters were calculated from Nyquist plots recorded at −0.8 V applied voltage

Photoanode	*R*_S_ (Ohm cm^2^)	*R*_CT_ (Ohm cm^2^)	Chemical capacitance (F)	Electron lifetime *τ*_n_ (ms)	Electron transport lifetime *τ*_n_ (ms)	Charge collection efficiency (%)
FTO/TiO_2_/NSCNSs/PTB7-Th:PC71BM/MoO_3_/Ag	29.97	144.47	3.01 × 10^−4^	41.36	43.49	82.81
FTO/TiO_2_/NSCNSs/RuQDs/PTB7Th:PC71BM/MoO_3_/Ag	24.37	246.37	3.57 × 10^−4^	85.61	87.96	91.01

The addition of ruthenium quantum dots in the light harvesting layer has resulted in an increase in the short circuit current density (*J*_SC_) and the performance of OSCs. Moreover, as seen in Fig. S8(a), the chemical capacitance of the BHJ device containing the light-absorbing layer of NSCNSs/RuQDs (which have relatively low surface area and porosity) increases with the increase in applied potential towards the open circuit condition.^61,62^ On the other hand, the chemical capacitance of the BHJ device containing the light-absorbing layer of NSCNSs (which have relatively high surface area and porosity) decreases with the increase in applied potential.^63–65^ The possible reason behind the above-mentioned unusual behavior can be the decreased role of double layer capacitance in the total chemical capacitance with the rise in applied potential.^66^ As compared to the NSCNSs and NSCNSs/RuQDs light-absorbing layers, the former has slightly smaller crystal sizes, pore sizes, and pore diameters owing to the doping effect of Ru. The latter facilitates improved charge transport within the TiO_2_/NSCNSs/RuQDs active layer, resulting in more efficient charge separation and collection in the organic solar cell system.

Hence, the devices made from the BHJ configuration, namely FTO/TiO_2_/NSCNSs/PTB7-Th:PC71BM/MoO_3_/Ag and FTO/TiO_2_/NSCNSs/RuQDs/PTB7-Th:PC71BM/MoO_3_/Ag, have different chemical capacitances. It should be noted that the FTO/TiO_2_/NSCNSs/RuQDs/PTB7-Th:PC71BM/MoO_3_/Ag BHJ device has higher chemical capacitance as compared to the other two devices. This is clearly visible in Fig. S8(a) at −0.8 V applied potential, which is near the open-circuit condition. Therefore, the device with BHJ structure FTO/TiO_2_/NSCNSs/RuQDs/PTB7-Th:PC71BM/MoO_3_/Ag.

The process of electron recombinations occurring at the (FTO/TiO_2_/NSCNSs/RuQDs) electron transport layer and active layer interfaces may be quantified by the recombination lifetime (*τ*_n_), as defined in the following eqn (3):^67^

3 *τ*_n_ = *R*_ct_·*C*_µ_

Herein, *R*_ct_ represents the charge-transfer resistance, while *C*_µ_ corresponds to the chemical capacitance of the FTO/TiO_2_/NSCNSs/RuQDs and PTB7-Th:PC71BM interface, respectively. Further, EIS parameters listed in Table 2 have been used to determine the charge collection efficiency (*η*_CC_) of the bulk-heterojunction (BHJ) device in organic solar cells (OSCs) by means of the following relationship:^68,69^

4 *η*_CC_ = (1 + *R*_s_/*R*_ct_)^−1^

Fig. S8(b) and Table 2 show the electron transport lifetimes (*τ*_n_) for all BHJ devices. It can be seen that for the FTO/TiO_2_/NSCNSs/PTB7-Th:PC71BM/MoO_3_/Ag configuration, *τ*_n_ is 43.49 ms, but in the case of the FTO/TiO_2_/NSCNSs/RuQDs/PTB7-Th:PC71BM/MoO_3_/Ag configuration, *τ*_n_ is 87.96 ms. Obviously, in the measurements, the longer electron lifetime has been found in the case of FTO/TiO_2_/NSCNSs/RuQDs/PTB7-Th:PC71BM/MoO_3_/Ag device. In such a way, the FTO/TiO_2_/NSCNSs/RuQDs/PTB7-Th:PC71BM/MoO_3_/Ag BHJ device acting as an ETL exhibits better electron transport than FTO/TiO_2_/NSCNSs/PTB7-Th:PC71BM/MoO_3_/Ag BHJ devices. In addition, the *τ*_n_ values obtained based on EIS parameters reveal a slower process of electron recombination in the FTO/TiO_2_/NSCNSs/RuQDs/PTB7-Th:PC71BM/MoO_3_/Ag BHJ device. Such a phenomenon has been explained by the presence of NSCNSs/RuQDs in the ETLs, which promotes better charge transport and leads to an increase in the electron diffusion length. The decreasing order of electron lifetimes (*τ*_n_) for all investigated OSCs depending on the FTO/TiO_2_/NSCNSs/RuQDs/PTB7-Th:PC71BM/MoO_3_/Ag configurations in OSCs at bias −0.8 V is presented in Fig. 8(d). As seen from Fig. 8(d), in the case of the FTO/TiO_2_/NSCNSs/RuQDs/PTB7-Th:PC71BM/MoO_3_/Ag configuration, the longest electron–electron lifetime has been observed. This fact confirms the idea that such a bilayer structure provides OSCs with the best photovoltaic performance. In addition, the results shown in Fig. S8(c) demonstrate that the higher value of the charge collection efficiency (*η*_CC_), calculated according to EIS parameters, is found in FTO/TiO_2_/NSCNSs/RuQDs/PTB7-Th:PC71BM/MoO_3_/Ag. This improvement highlights the beneficial effect of integrating Ru quantum dots (RuQDs) as an ETL together with light-harvesting nanostructures in the BHJ active layer. According to Table 2 and Fig. S8(c), the FTO/TiO_2_/NSCNSs/RuQDs/PTB7-Th:PC71BM/MoO_3_/Ag BHJ device-based OSCs exhibit a significantly higher *η*_CC_ charge collection efficiency (91.01%) compared to the FTO/TiO_2_/NSCNSs/PTB7-Th:PC71BM/MoO_3_/Ag BHJ device (82.81%) based OSCs. These findings suggest that the slower recombination process and enhanced charge collection efficiency of the OSCs fabricated using the FTO/TiO_2_/NSCNSs/RuQDs/PTB7-Th:PC71BM/MoO_3_/Ag device ETLs contribute to improved *J*_SC_ values compared to other BHJ device-based OSCs.

## 4 Conclusions

In summary, a quantum-confinement and defect-engineered two-dimensional porous NSCNSs/RuQDs hybrid nanostructure was successfully fabricated *via* a facile solvothermal strategy and employed as an efficient electron transport layer for inverted organic solar cells. The integration of ultrasmall Ru quantum dots within the N,S-codoped carbon nanosheet framework establishes a strongly coupled metal–carbon interface with interconnected charge-transport pathways, enabling simultaneous regulation of electronic structure, defect chemistry, and interfacial carrier dynamics. Comprehensive structural and spectroscopic analyses reveal that RuQD incorporation promotes charge redistribution, defect modulation, and quantum-confinement effects within the carbon framework. The resulting NSCNSs/RuQDs hybrid exhibits enhanced visible-to-near-infrared absorption, reduced optical bandgap from 0.87 to 0.34 eV, and improved carrier transport properties. The prolonged photoluminescence lifetime from 1.73 to 5.44 ns demonstrates effective suppression of nonradiative recombination through Ru-mediated defect passivation and enhanced interfacial charge separation. When applied as a TiO_2_/NSCNSs/RuQDs bilayer electron transport layer in PTB7-Th:PC71BM-based inverted OSCs, the hybrid interface facilitates efficient electron extraction, reduced transport losses, and improved charge collection, achieving a PCE of 10.77 ± 0.57% with *J*_SC_ = 23.18 ± 0.51 mA cm^−2^, *V*_OC_ = 0.83 ± 0.01 V, and FF = 0.56 ± 0.01. EIS analysis further confirms prolonged electron lifetime and enhanced charge collection efficiency (91.01%), indicating that the improved device performance originates from accelerated carrier transport and suppressed interfacial recombination. This work provides a versatile strategy for integrating quantum-confined metal nanostructures with heteroatom-engineered carbon frameworks to develop multifunctional interfacial materials for next-generation photovoltaic and optoelectronic applications.

## Author contributions

B. Murali: writing – original draft, writing – review and editing, methodology, investigation, analysis, data curation, conceptualization, project administration, software. Thamilselvan. A: analysis, data curation, conceptualization, writing – review and editing. Tholkappiyan. Ramachandran: investigation, data curation, conceptualization, writing – review and editing. S. Sathiyaraj: investigation, data curation, conceptualization, writing – review and editing. Shanmugavel. Chinnathambi: investigation, analysis, data curation, conceptualization, writing – review and editing. Natarajan. Arumugam: investigation, data curation, conceptualization, writing – review and editing. Mohammad. Ahmad. Wadaan: investigation, data curation, conceptualization, writing – review and editing. Sandhanasamy. Devanesan: investigation, data curation, conceptualization, writing – review and editing.

## Conflicts of interest

The authors declare that they have no known competing financial interests or personal relationships that could have appeared to influence the work reported in this paper.

## Supplementary Material

RA-OLF-D6RA06453A-s001

## Data Availability

All data supporting the findings of this study are available from the corresponding authors upon reasonable request. Supplementary information (SI): experimental procedures, synthesis and device fabrication protocols, materials, supplementary characterization data (HRTEM, EDS, and optical bandgap analysis), photovoltaic performance statistics (*n* = 7), and electrochemical impedance spectroscopy (EIS) data, including equivalent-circuit fitting, chemical capacitance, electron transport lifetime, and charge-collection efficiency analyses. See DOI: https://doi.org/10.1039/d6ra06453a.
