# Supplementary material for: Quantum-confinement and defect-engineered N,S-carbon nanosheet–ruthenium quantum dot heterostructures for highly efficient organic solar cells
Source: RSC Adv. 2026 Aug 11;16(40):46553–70. doi: 10.1039/d6ra06453a (PMC13458450; doi:10.1039/d6ra06453a)

Supplementary Information (SI) for RSC Advances.
This journal is © The Royal Society of Chemistry 2026

Supporting Information

Quantum-Confinement and Defect-Engineered N, S-Carbon Nanosheet–Ruthenium Quantum Dot Heterostructures for high-Efficient Organic Solar Cells

Murali Balu[1,*], Thamilselvan A[2], Tholkappiyan Ramachandran[3,*], S. Sathiyaraj[4], Shanmugavel Chinnathambi[1], Natarajan Arumugam[5], Mohammad Ahmad Wadaan[6], Sandhanasamy Devanesan[6]

[1]*Centre for Applied Nanomaterials, Chennai Institute of Technology, Kundrathur, Chennai – 600 069, India.*

[2]*Basic Sciences-Chemistry, SR University, Telangana-506371, India.*

[3]*Department of Physics, College of Science, United Arab Emirates University, Al-Ain, Abu Dhabi, P. O. Box 15551, United Arab Emirates.*

[4]*Department of Mechanical Engineering, Aarupadai Veedu Institute of Technology, Vinayaka Missions Research Foundation (DU), Chennai. 603104*

[5]*Department of Chemistry, College of Science, King Saud University, P.O. Box 2455, Riyadh 11451, Saudi Arabia.*

[6]*Bioproducts Research Chair, Department of Zoology, College of Science, King Saud University, P.O. Box 2455, Riyadh, 11451, Saudi Arabia.*

****Corresponding author****: muralib374@gmail.com; Tel: +918124715364*
thols2006@gmail.com

Experimental section

1.1. Materials

All solvents used in this work were of HPLC grade and employed without further purification. Commercially available TiO_2 anatase nanopowder (particle size ≈25 nm, 99.7% purity), citric acid monohydrate ($C_6H_8O_7\cdot H_2O$), thiourea (CH_4N_2S), ruthenium(III) chloride hydrate ($RuCl_3\cdot xH_2O$), N,N-dimethylformamide (DMF), PTB7-Th:$PC_{71}BM$, and 1,2-

dichlorobenzene/N-methyl-2-pyrrolidone (NMP) solvent mixture were purchased from Sigma-Aldrich (India) and used as received without further purification. Fluorine-doped tin oxide (FTO) glass substrates (TEC15, 2.2 mm thickness) were purchased from Greatcell Solar (Dyesol).

1.1. Preparation of NSCNSs paste

The following procedure was adopted in the preparation of the NSCNSs scattering layer paste; 1 gram of the dry NSCNSs nanopowder was mixed with 0.3 grams of ethyl cellulose, 2 milliliters of terpineol, 1 milliliter of acetic acid, and 10 milliliters of ethanol in an Erlenmeyer flask. The mixture was then sonicated for one hour to yield a smooth white suspension, which was stirred magnetically for 24 hours at a speed of 300 rpm.

1.2. Preparation of NSCNSs/RuQDs paste

The paste for preparation of the NSCNSs/RuQDs scattering layer was prepared using the same procedure; however, 1 gram of the dried NSCNSs/RuQDs nanocomposite powder was mixed with 0.3 grams of ethyl cellulose along with 2 milliliters of terpineol, 1 milliliter of acetic acid, and 10 milliliters of ethanol in an Erlenmeyer flask. This was done by sonication for 1 hour followed by magnetic stirring at 300 rpm for 24 hours.

2. Characterization methods

The crystal structures and phases of porous NSCNSs and NSCNSs/RuQDs were investigated using Rigaku Ultima IV X-ray diffractometry (XRD). The step size used during scanning was 0.02 s, and the Cu-Kα source with a wavelength of 0.154 nm was applied. Morphologies of the porous NSCNSs and NSCNSs/RuQDs samples were studied using HRSEM equipment (Thermo Fisher Scientific Apreo S). Analyses of HRTEM were carried out with a JEOL JEM-2100 Plus microscope (Japan). The absorbance and diffuse reflectance spectra of samples were collected in the 200-1400 nm range using a Shimadzu UV-3600 I Plus UV-vis-NIR spectrometer. Surface groups were identified using FT-IR spectroscopy in the 400-4000 cm^{-1} range on a Shimadzu IR Tracer-100 spectrometer. PL spectra of samples were recorded with an Agilent Cary Eclipse Fluorescence Spectrophotometer (model G9800B). Chemical compositions and oxidation states of surface elements were measured by XPS analysis on a PHI Versa Probe III microprobe, followed by the curve fitting and peak deconvolution of each individual signal. Photovoltaic properties of OSCs were estimated by measuring their current density-voltage (J-V) characteristics using a Keithley 2400 source

meter under simulated AM1.5G solar illumination emitted from a Newport Sun solar simulator. The area illuminated was limited by a square aperture with an area of 0.20 cm^2, which corresponded to the active layer area of the devices. EIS measurements were performed on a CH Instruments electrochemical analyzer (Model 604E, USA) with the frequency range of 100 kHz to 0.1 Hz and the bias sweep range of -0.9 V with a 10 mV amplitude. The impedance curves of all BHJ OSCs were analyzed using relevant equivalent circuit models in conjunction with Z-view software.

3. Fig. S1: Schematic illustration of the synthesis of porous N, S-codoped Carbon Nanosheets (NSCNSs).

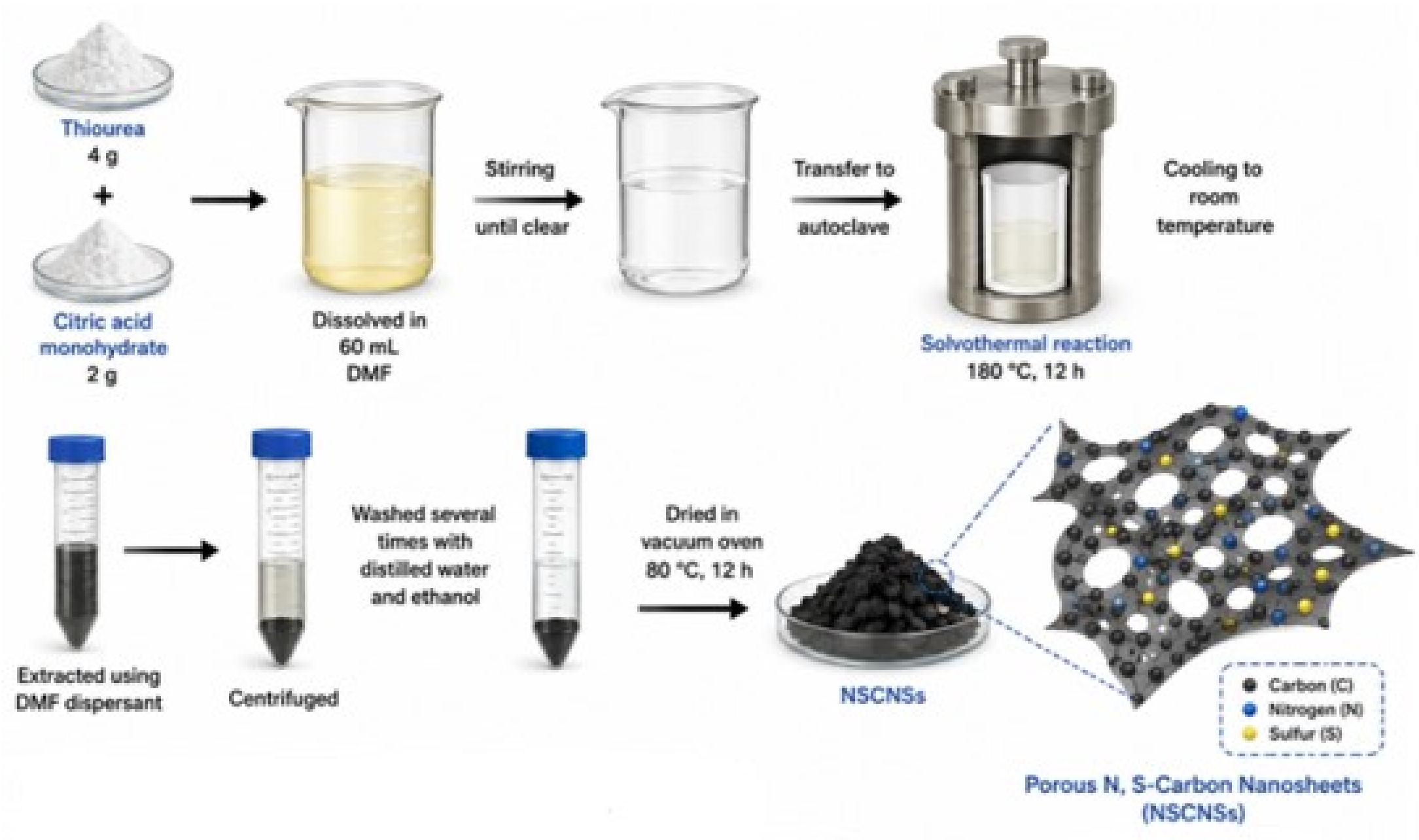

4. Fig. S2: Schematic illustration of the synthesis of porous N, S-Carbon Nanosheets and Ruthenium Quantum Dots (NSCNSs/RuQDs) Hybrid Structure

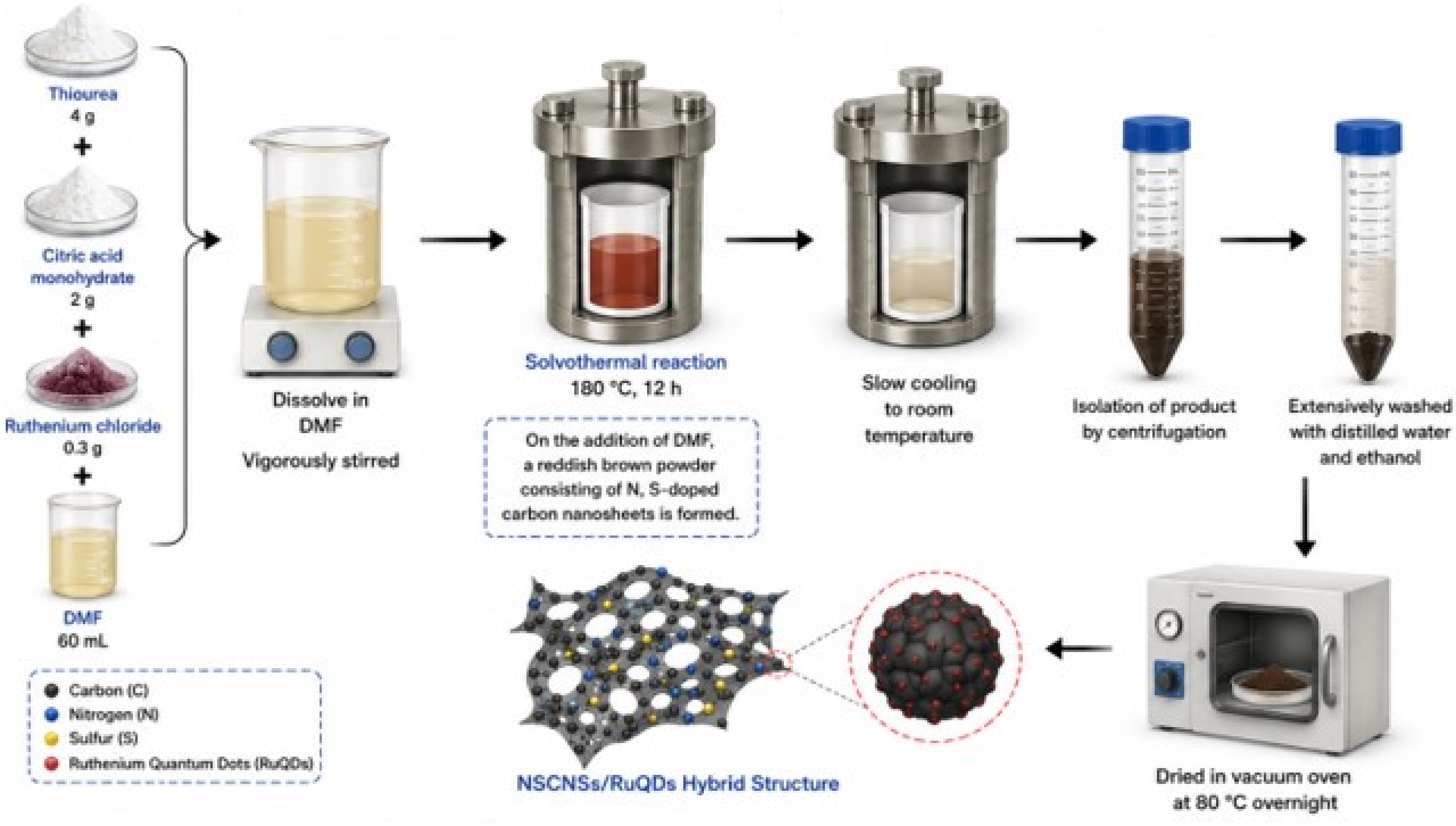

5. Fig. S3: The EDS spectrum of HRTEM images (a) NSCNSs, and (b) NSCNSs/RuQDs hybrid structure.

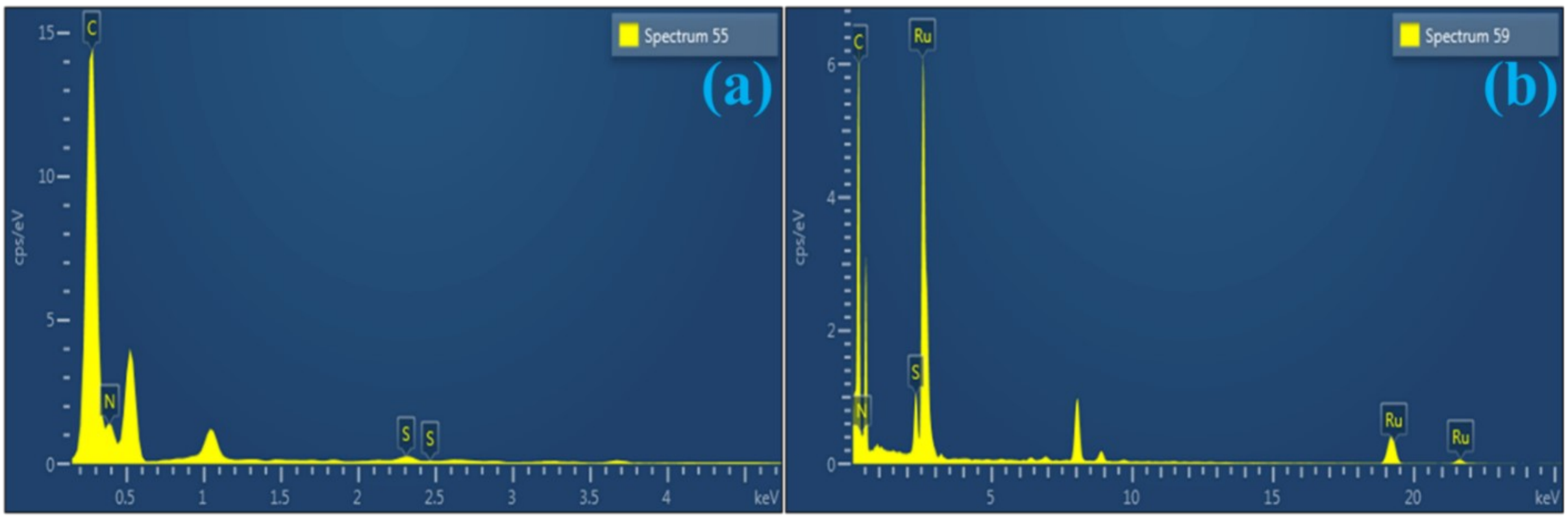

6. **Fig. S4: Tauc plots $(\alpha h\nu)^{1/2}$ versus photon energy for (a) NSCNSs/RuQDs and (b) pristine NSCNSs. The optical bandgaps were estimated by linear extrapolation of the absorption edge, yielding values of 0.34 eV (R^2 = 0.95) for the NSCNSs/RuQDs hybrid and 0.87 eV (R^2 = 0.98) for the pristine NSCNSs.**

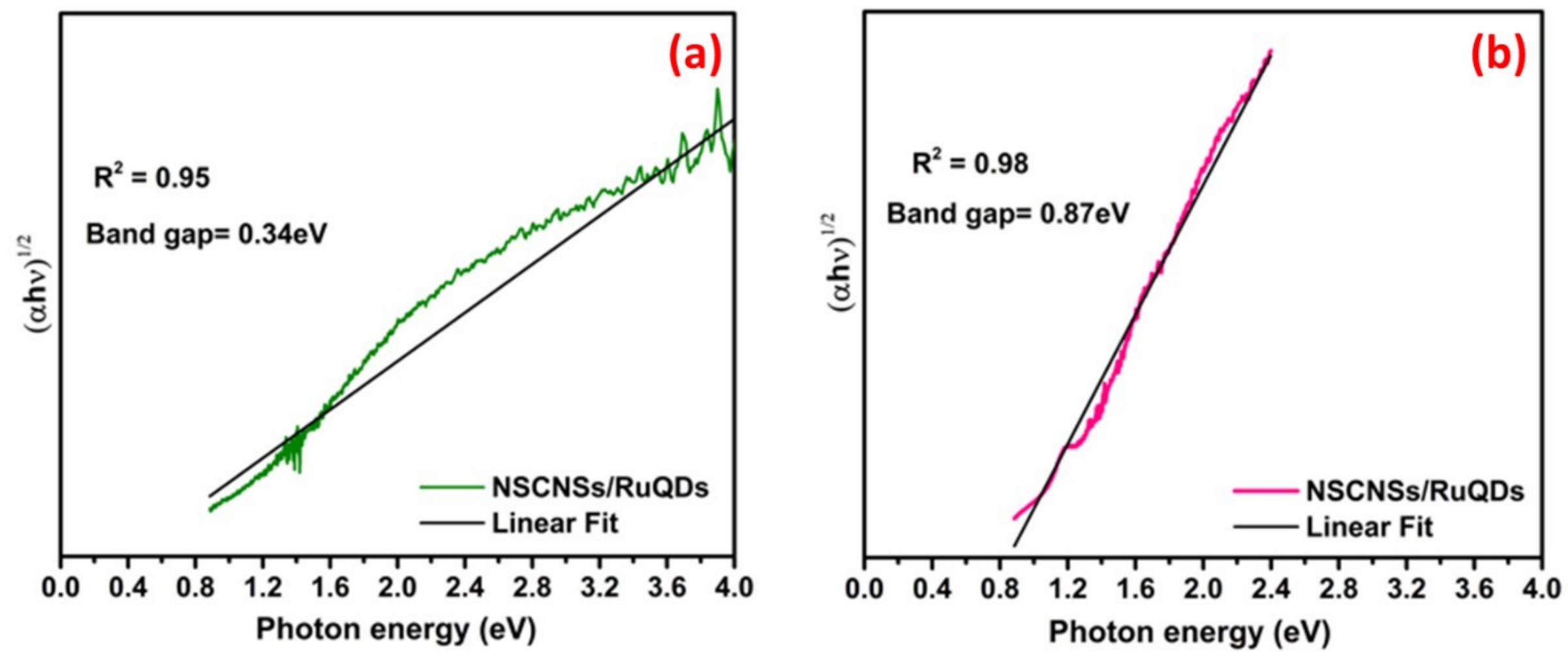

7. **Fig. S5: Fabrication of OSCs using porous NSCNSs and NSCNSs/RuQDs hybrid nanostructures as the electron transport layer.**

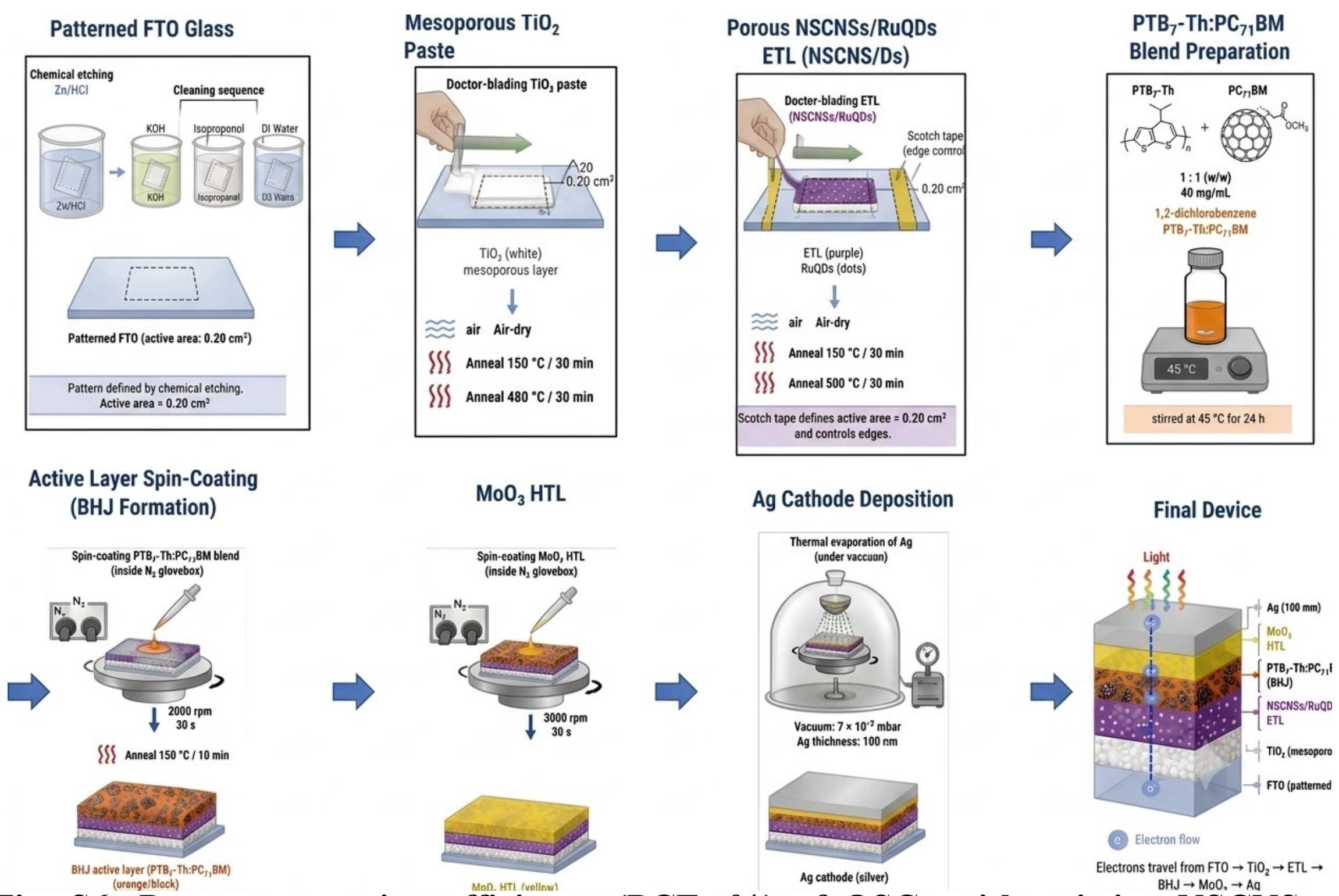

8. **Fig. S6: Power conversion efficiency (PCE, %) of OSCs with pristine NSCNSs and**

NSCNSs/RuQDs electron transport layers (ETLs) in bulk-heterojunction (BHJ) devices. Error bars represent one standard deviation (n = 7).

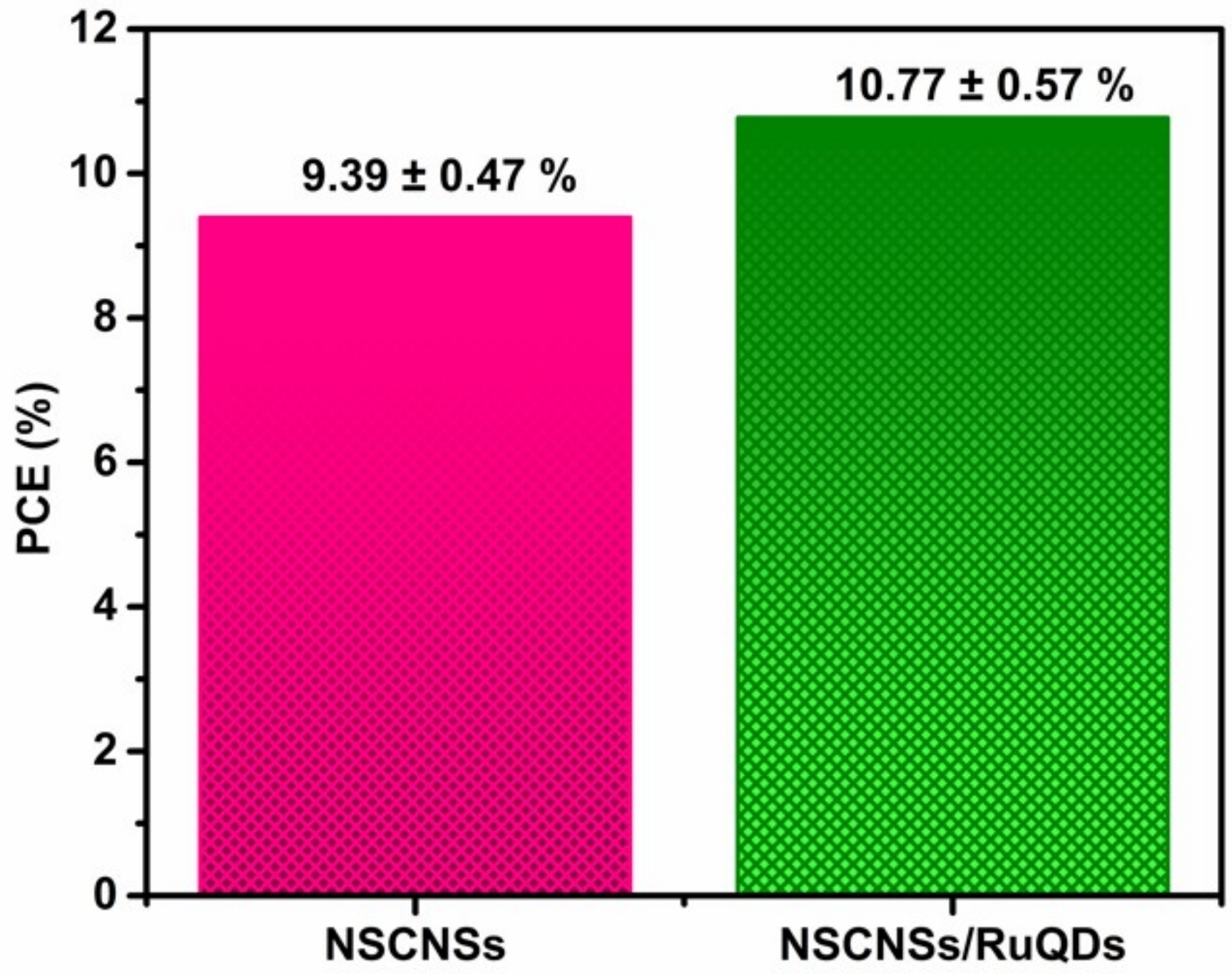

9. Fig. S7: EIS parameters of organic solar cells obtained from the Nyquist plots were recorded at an applied bias potential of -0.8 V, and the obtained data were fitted with an equivalent circuit model.

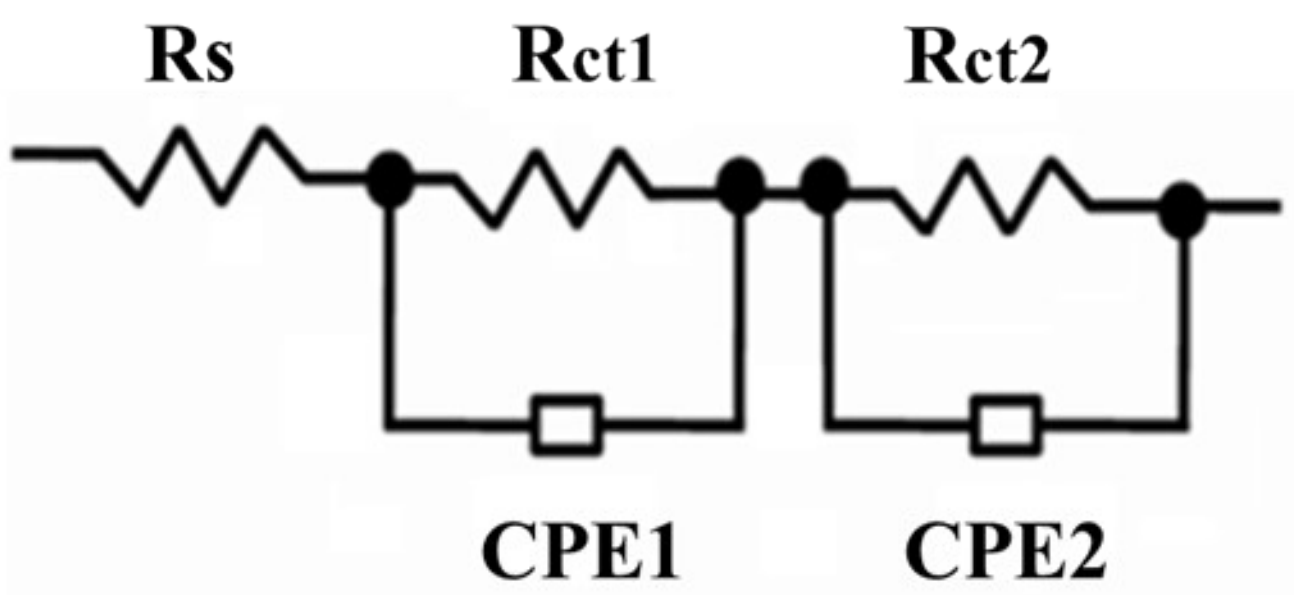

10. Fig. S8: Electrochemical impedance spectroscopy (EIS) data deduced results of (a) chemical capacitance, (b) Electron transport lifetime, and (c) Charge collection efficiency (%) of BHJ FTO/TiO_2/NSCNSs/PTB7-Th:PC71BM/MoO_3/Ag, and FTO/TiO_2/NSCNSs/RuQDs/PTB7-Th:PC71BM/MoO_3/Ag device with different light absorbing nanostructures at (-0.8 V) applied bias potentials (recorded under dark).

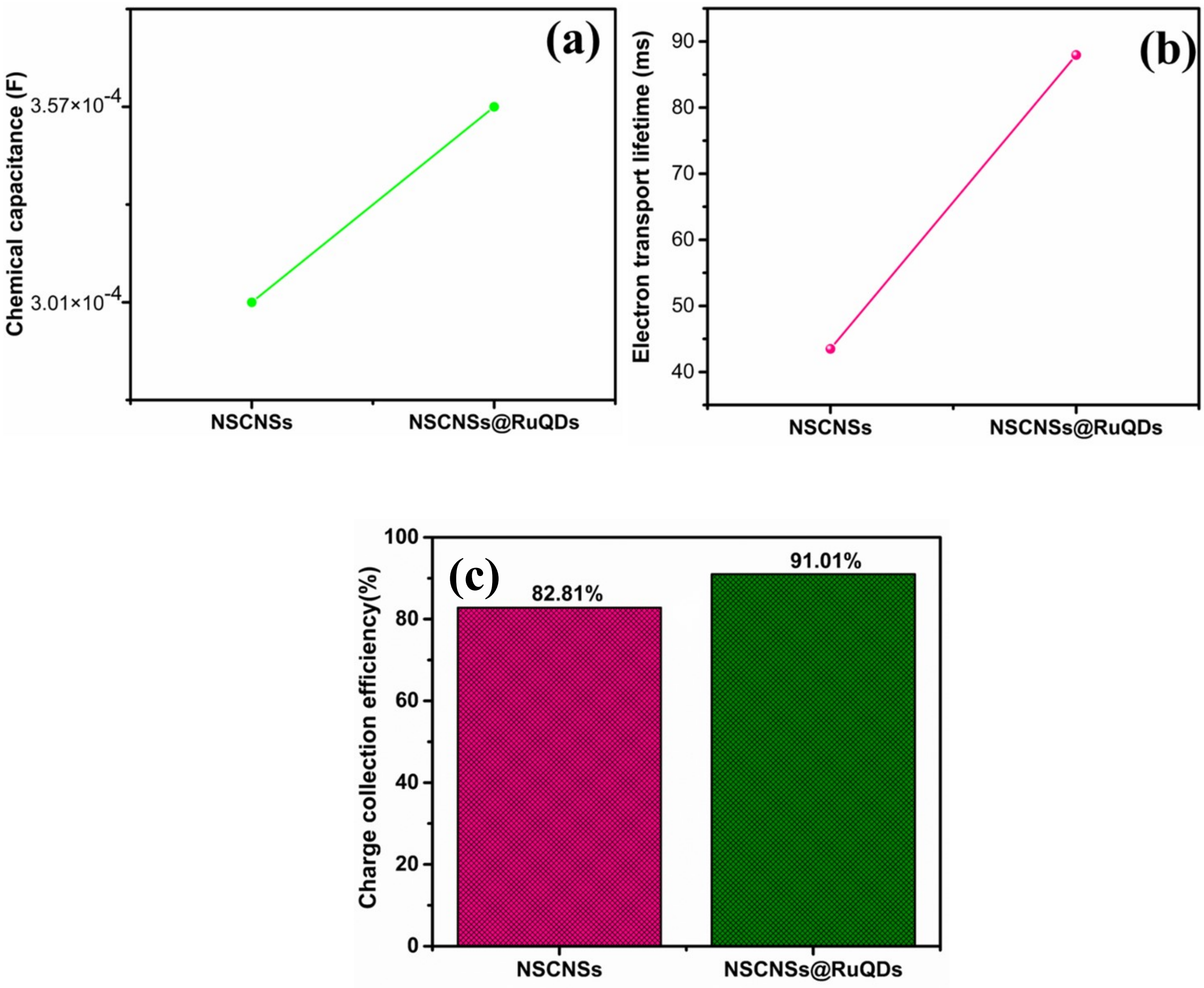

Supplement: Supplementary file 1 [file RA-016-D6RA06453A-s001.pdf]
